# The Impact of Methanol Concentration on Recombinant Protein Glycosylation in *Pichia pastoris* SuperMan5

**DOI:** 10.1111/1751-7915.70272

**Published:** 2025-12-05

**Authors:** Andre Ohara, Zhang Pengyue, Xinqi Cao, Roberto Donini, Stuart M. Haslam, Karen M. Polizzi

**Affiliations:** ^1^ Department of Chemical Engineering Imperial College London London UK; ^2^ Imperial Centre for Engineering Biology Imperial College London London UK; ^3^ Department of Life Sciences Imperial College London London UK

**Keywords:** bioprocess optimization, glycosylation modulation, *Pichia pastoris*, post‐translational modifications, recombinant protein production

## Abstract

The methylotrophic yeast *Pichia pastoris* (also known as *Komagataella phaffii*) is a prominent platform for recombinant protein production, offering benefits such as thermo‐ and osmotolerance, high‐density growth, and efficient protein secretion. Its ability to metabolise methanol, an increasingly available carbon source, enhances its cost‐effectiveness and sustainability for industrial use. As a eukaryotic host, *P. pastoris* ensures proper protein folding and post‐translational modifications (PTMs), including glycosylation, which is essential for correct folding and endoplasmic reticulum (ER) quality control. While ER‐transferred glycans are critical for maturation, additional modification in the Golgi apparatus can yield larger glycans whose impact on stability, solubility, and bioactivity may be either beneficial or undesirable, depending on the application of the heterologous protein. The impact of induction conditions on glycosylation of proteins secreted by *P. pastoris* SuperMan5 was examined, using the DS‐1 (G2P[4]) and WA (G1P[8]) VP8* rotavirus capsid proteins as a model. An ELISA‐based screening system was employed for clone selection and media optimization, with results showing easy integration into automated workflows. Methanol concentration was found to impact both *N*‐ and *O‐*linked glycosylation complexity, shaping the glycosylation profile of the target protein as well as the *P. pastoris* secretome. This study underscores the importance of optimising cultivation conditions to enhance protein yield, refine glycosylation, and minimise impurities, all of which are crucial for large‐scale production and efficient downstream processing. It also suggests a method for easy modulation of glycosylation depending on the target application and the desired level of glycosylation.

## Graphical abstract

The impact of methanol concentration during induction on N‐ and O‐linked glycosylation profiles was studied in a glycoengineered *Pichia pastoris* strain. Higher methanol concentrations led to higher mannose glycans suggesting that this variable can be used to manipulate protein quality during bioprocessing.

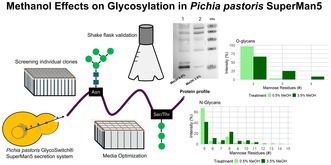

## 1 Introduction

The methylotrophic yeast *Pichia pastoris* (syn. *Komagataella phaffii*) has become the second most widely used platform for recombinant protein production, after *Escherichia coli* , and is the most commonly used eukaryotic host, surpassing other conventional and non‐conventional yeasts (Ergün et al. 2021). *P. pastoris* offers advantages such as thermo‐ and osmotolerance (Dragosits et al. 2010; Lin et al. 2021), robust respiratory growth at extremely high cell densities (Duman‐Özdamar and Binay 2021), efficient protein secretion (Damasceno et al. 2012), and the ability to express recombinant proteins at elevated levels using both strong constitutive and inducible promoters (Kaushik et al. 2020).

The ability to use methanol as a sole carbon and energy source as well as an inducer for protein expression establishes *P. pastoris* as a cost‐effective and sustainable host that can use one‐carbon (C1) feedstock for industrial‐scale protein production (Meng et al. 2023; Orsi et al. 2023; Srivastava et al. 2022). Methanol has a strong reducing potential and is increasingly available from renewable methods such as CO_2_ hydrogenation or methane‐to‐methanol bioconversion. This approach offers an efficient fermentation system with rapid production, minimal space needs, and independence from environmental variation (Knychala et al. 2024; Ritala et al. 2017).

As a eukaryotic host, *P. pastoris* possesses an endoplasmic reticulum (ER) and Golgi apparatus that ensure protein folding quality control and enable post‐translational modifications (PTMs), such as glycosylation, the covalent attachment of sugar molecules to proteins or lipids (Vorauer‐Uhl and Lhota 2019). In yeast, these modifications include *N*‐linked glycosylation on asparagine residues in the Asn‐Xaa‐Thr/Ser consensus sequence and *O*‐linked glycosylation on threonine or serine hydroxy groups (Bretthauer and Castellino 1999).

Protein glycosylation, essential for glycoprotein structure, solubility, stability, and bioactivity, depends on factors such as the host, recombinant protein, and culture conditions (Karengera et al. 2017; Pekarsky et al. 2018). *N*‐glycosylation enhances glycoprotein secretion in *P. pastoris*, though outcomes vary depending on glycosylation site positioning and remain poorly understood (Wang et al. 2022). *O*‐glycosylation in *P. pastoris* is far less characterised with reports of short *O*‐glycans containing up to 3–6 mannose residues, contrasting with longer *O*‐glycans in *Candida albicans* (5‐Man) and *S. cerevisiae* (13‐Man) (Radoman et al. 2021).

In recent years, glycoengineered *P. pastoris* strains—such as the *Pichia GlycoSwitch SuperMan5* series (M5, M5GN, M5GNGal, M3GN, and M3GNGal; BioGrammatics Inc.)—have been developed to produce recombinant proteins with more uniform and human‐like glycosylation patterns, making them preferred host cells for therapeutic protein expression. In the M5 (GS 10010) strain used in the present study, the *OCH1* gene, which encodes the α‐1,6‐mannosyltransferase responsible for elongation of glycan structures in the Golgi apparatus, is disrupted. Additionally, expression of an ER‐resident α‐1,2‐mannosidase trims glycans to a consistent Man_5_GlcNAc_2_ structure (Effenberger et al. 2017). This strain has become particularly interesting for the development of biopharmaceuticals, where the precise control of glycosylation patterns is crucial for reducing immunogenicity and improving therapeutic efficacy (Wolf et al. 2022). SuperMan5 has successfully been used in the expression of therapeutics such as the Human Chymase (EC 3.4.21.39) (Smith et al. 2014), and proinsulin (Baeshen et al. 2016).

Glycosylation requirements are determined by the physical, chemical, and biological properties of the target product. For example, (Dalvie et al. 2021), strategically removed predicted glycosylation sites from rotavirus capsid protein to prevent hypermannosylation, thereby simplifying chromatographic separation. This optimization facilitated the co‐expression of three antigens and enabled their purification within a single production cycle. Conversely, the addition of *N*‐glycosylation motifs has been shown to enhance foaming, emulsifying properties, and gel water‐holding capacity of Patatin (Tao et al. 2024), improve Elastase stability in solvents (Han et al. 2014), and maintain the stability of β‐glucuronidase under denaturing conditions (Zou et al. 2013).

Motivated by the specific requirements for glycosylation—critical factors that significantly influence its functionality and application—we have devised a straightforward yet effective method to modulate glycosylation in proteins secreted by *P. pastoris* SuperMan5, using the DS‐1 (G2P[4]) and WA (G1P[8]) VP8* rotavirus capsid proteins as a model. By varying methanol concentrations across microscale and shake flask expression, we examined both *N*‐ and *O*‐glycosylation patterns in the target protein under different conditions. Recognising that these modifications may be advantageous or undesirable depending on the application, our primary goal was to develop a scalable workflow for fine‐tuning glycosylation patterns, unlocking broad potential across diverse biotechnological industries.

## 2 Material and Methods

### 2.1 Microbial Host Strain and Plasmids

*E. coli* DH5α (NEB 5‐α) was sourced from New England Biolabs (Hertfordshire, UK), while the humanised glycoengineered *P. pastoris* strain Glycoswitch SuperMan5‐10 GS 10010 (Genotype: och1‐∆1, GAP‐mannosidaseHDEL) was obtained from Biogrammatics Inc., USA. The integrative plasmid pD912‐AK was provided by Atum Inc. (formerly DNA 2.0). The DS‐1 (G2P[4]) and WA (G1P[8]) VP8* sequences, were codon optimised for *P. pastoris* and synthesised by GeneArt Gene Synthesis (Thermo Fisher Scientific). Details of all constructed plasmids, primers, and the yeast strain construction workflow are presented in Appendix S1.

### 2.2 Screening of *P. pastoris* Clones Expressing DS‐1 (G2P[4]) VP8*

The screening process was conducted following the approach described by Kaushik et al. (2020), with minor modifications. A sterile 96‐well U‐bottom deep‐well plate (DWP) was prepared by adding 500 μL of YPD medium (1% yeast extract, 2% peptone, 2% dextrose) to each well. Individual colonies (20 clones) of *P. pastoris* transformed with the DS‐1 (G2P[4]) or WA (G1P[8]) VP8* construct were inoculated, alongside duplicate wells inoculated with a glycerol stock of *P. pastoris* GlycoSwitch SuperMan5 carrying the empty vector pD912‐AK as a negative control. The plate was incubated at 30°C with shaking (1000 rpm, 3 mm throw diameter) in a VorTemp 56 Shaking Incubator for 48 h.

From this pre‐culture, 50 μL aliquots were transferred into a fresh sterile plate containing 600 μL of BMGY medium (1% yeast extract, 2% peptone, 100 mM potassium phosphate buffer, pH 6.0; 1.34% yeast nitrogen base; 0.4 μg/mL biotin; 1% glycerol) per well, followed by incubation at 30°C for an additional 48 h. Meanwhile, the remaining pre‐culture was supplemented with an equal volume of 50% glycerol (final concentration 25% v/v), sealed, and stored at −80°C as a glycerol stock.

After 48 h, cells were harvested by centrifugation at 4000 ***g*** for 5 min and resuspended in 300 μL of BMM medium (100 mM potassium phosphate buffer, pH 6.0; yeast nitrogen base; 0.4 μg/mL biotin; 2% methanol) to induce the AOX1 promoter. The cultures were incubated at 21°C for a further 48 h. Post‐induction, clarified supernatants were analysed via Western blot and immunoassays to determine the secretion levels of DS‐1 (G2P[4]) VP8*.

The top‐performing clone was selected, and its genomic DNA was extracted following (Moreno et al. 1991) for subsequent determination of the integrated gene copy number via whole‐genome sequencing using nanopore technology (Full Circle Labs Ltd).

### 2.3 Indirect Enzyme‐Linked Immunosorbent Assay (ELISA)

The clarified supernatants were diluted 1:5 in 50 mM carbonate–bicarbonate buffer (pH 9.6), and 100 μL of the solution was added to the wells of a Maxisorp polystyrene plate, followed by overnight incubation at 4°C. The plates were blocked at room temperature for 1 h with 200 μL of blocking buffer (PBS‐T 0.05% + 5% skim milk) per well.

After blocking, 100 μL of either anti‐6 × His‐tag mouse monoclonal antibody (1:1000 dilution, J095G46, BioLegend) or sheep polyclonal rotavirus antibody (1:1000 dilution, ab252741, Abcam, UK), diluted in blocking buffer, was added and incubated for 1 h at room temperature with gentle agitation. The plates were washed three times with PBS‐T (0.05%), followed by the addition of 100 μL of either goat anti‐mouse IgM‐AP (1:5000 dilution, A16069, Thermo Fisher Scientific) or donkey anti‐sheep (H + L)‐AP secondary antibody (1:5000 dilution, A16044, Thermo Fisher Scientific), diluted in blocking buffer. This was incubated for 30 min at room temperature. After four washes, colour development was initiated with 100 μL of 1‐Step PNPP Substrate Solution (Thermo Fisher Scientific), yielding a yellow, water‐soluble product. Absorbance was measured at 405 nm.

### 2.4 Kex2 Cleavage, PNGaseF Treatment and Western Blot Analysis

The Kex2 protease reaction was carried out by mixing 10 μL of culture supernatant, 9.5 μL of buffer (50 mM Tris–HCl, 5 mM CaCl₂, pH 7.0), and 0.5 μL of Kex2 enzyme (1 mg/mL, Abcam, ab96554). The reaction was gently mixed and incubated overnight at 37°C. PNGaseF (New England Biolabs) was used according to the manufacturer's protocol, with the modification that samples were incubated for 4 h at 37°C before Western blot processing. Sodium dodecyl sulfate‐polyacrylamide gel electrophoresis (SDS‐PAGE) was performed on 16% gels. *P. pastoris* culture supernatants were denatured by boiling for 5 min in reducing SDS sample buffer (0.0625 M Tris–HCl, pH 6.8, 2.3% w/v SDS, 10% w/v glycerol, 0.01% w/v Bromophenol Blue, 0.5 mM DTT).

Molecular weights were estimated using the prestained protein ladder, Fisher BioReagents (10–170 kDa, Thermo Fisher Scientific). Proteins were transferred to a polyvinylidene fluoride (PVDF) membrane (Merck Millipore) using a Mini‐PROTEAN (Bio‐Rad) electrophoresis cell. Detection of DS‐1 (G2P[4]) VP8* and WA (G1P[8]) VP8* was performed using an anti‐6 × His‐tag mouse monoclonal antibody (1:1000 dilution) (BioLegend) followed by a secondary goat anti‐mouse IgM‐AP antibody (1:5000 dilution). Bands were visualised using the BCIP/NBT alkaline phosphatase substrate kit (Thermo Fisher Scientific).

### 2.5 Activity and Expression of α‐Mannosidase in Cell Lysates

Cell pellets from three methanol induction conditions (0.00%, 0.50%, and 3.50%), each in triplicate biological replicates, were lysed following the methodology described in (Shivakumar et al. 2025). Protein content of the resulting lysates was quantified using the Pierce BCA Protein Assay Kit (Thermo Scientific), and α‐mannosidase activity (AMA) was assessed with the α‐Mannosidase Activity Assay Kit (Sigma‐Aldrich, MAK318). Specific AMA was expressed as Units L^−1^ mg^−1^ protein. To access the expression of the *T. reesei* 1,2‐α‐mannosidase tagged with an HDEL signal, western blot analysis was performed using an HDEL monoclonal antibody (1:1000; Thermo Fisher), followed by an anti‐mouse secondary antibody and BCIP chromogenic detection, as described above.

### 2.6 Microscale and Shake Flask Bioprocess With Methanol Supplementation

A starter culture was prepared by inoculating 5 mL of YPD medium with the strain glycerol stock and incubating it at 30°C with agitation (250 rpm) for 20 h. This culture was used to inoculate 100 mL of BMGY medium at an initial optical density (OD_600_) of 0.5, followed by incubation at 30°C with shaking (250 rpm) for 24 h, reaching an OD_600_ of 70. Cells were harvested by centrifugation at 1500 ***g*** for 5 min at room temperature, washed with sterile double‐distilled water, and resuspended in BMM medium to achieve OD_600_ values of 70 and 140, enabling a comparison between two cell densities for methanol induction. OD_600_ measurements were performed using an Eppendorf Biophotometer (Eppendorf).

The suspensions were distributed into the wells of a 24‐well deep‐well plate (DWP) in duplicate for each methanol concentration (0.0%, 0.5%, 1.0%, 1.5%, 2.0%, 2.5%, 3.0%, and 3.5%), and incubated at 20°C for 72 h. A negative control using *P. pastoris* GlycoSwitch SuperMan5 carrying the empty vector pD912‐AK was also included.

A similar approach was applied for shake flask cultivation, starting with a cell growth stage using glycerol as the main carbon source until an OD_600_ of 70 was reached. The resulting biomass was then concentrated to an OD_600_ of 140. For shake flask expression, 1 L baffled flasks containing 100 mL of concentrated cell resuspension in BMM were supplemented with methanol at concentrations of 0.5% or 3.5%. Induction was performed at 20°C with agitation at 250 rpm for 72 h.

### 2.7 Protein Purification and Buffer Exchange

Resin equilibration, sample preparation, and purification were performed using buffers prepared as follows: equilibration buffer (20 mM sodium phosphate, pH 8, 300 mM NaCl, 5% glycerol), wash buffer (same as equilibration buffer with 25 mM imidazole), elution buffer (same as equilibration buffer with 250 mM imidazole), and buffer exchange storage buffer (1× PBS, pH 7.4). A 100 mL *P. pastori*s culture was centrifuged at 3000 ***g*** for 15 min at 4°C, and the supernatant was adjusted to pH 8.0 with 2 M KOH. Pierce Protease Inhibitor Tablet, EDTA‐free (Thermo Fisher Scientific) was added to the clarified supernatant, followed by filtration through a 0.2 μm filter (Sartorius Minisart NML Plus).

The resin (1 mL bed volume of Ni‐NTA Agarose QIAGEN) was equilibrated by removing the storage buffer on a gravity stand, resuspending it in 10 mL of equilibration buffer, and centrifuging at 700 ***g*** for 5 min at 4°C. The clarified, pH‐adjusted sample was then added to the equilibrated resin and gently mixed at 4°C for 1 h to allow binding. Following incubation, the resin was centrifuged at 700 ***g*** for 5 min, and the supernatant was carefully discarded. The resin was resuspended in 10 mL of wash buffer, transferred to a gravity column (Pierce Disposable Columns—Thermo Fisher Scientific), and washed twice more with the same buffer. Finally, the bound proteins were eluted with 5 mL of elution buffer.

The eluate was immediately subjected to buffer exchange using an Amicon device (Amicon Ultra‐15 Centrifugal Filter Unit). Centrifugation was performed at 4000 ***g*** for 15 min at 4°C, with three sequential washes using 1× PBS pH 7.4 storage buffer to achieve buffer exchange. The protein concentration of purified samples was determined using the Pierce BCA Protein Assay Kit (Thermo Scientific). The final yields were calculated from the concentration and final volume of purified protein divided by the total volume of culture in the shake flask.

### 2.8 MALDI‐TOF MS Analysis

10 μg of purified DS‐1 (G2P[4]) VP8 samples from induction with 0.5% or 3.5% methanol, were reduced in 1 mL 0.6 M Tris buffer (pH 8.5) supplemented with 2 mg/mL DTT for 60 min at 50°C. Reduced samples were then carboxymethylated via the addition of 1 mL of 0.6 M Tris buffer (pH 8.5) supplemented with 12 mg/mL iodoacetic acid for 90 min at room temperature, in the dark. Samples were then dialysed against 5 × 4.5 L of 50 mM ammonium bicarbonate (pH 8.5) at 4°C and freeze dried after 48 h.

Lyophilized glycoproteins were then resuspended in 250 μL of 50 mM ammonium bicarbonate (pH 8.4) and tryptically digested with the addition of 10 μL of 1 mg/mL porcine pancreas trypsin (EC 3.4.21.4; Sigma‐Aldrich) in 50 mM ammonium bicarbonate buffer (pH 8.4) at 37°C for 24 h. After 24 h, the reaction was terminated with 3 drops of glacial acetic acid, and glycopeptides were purified using Oasis C_18_ HLB Plus 60 μm cartridges (Waters, USA) as described previously (North et al. 2010). Volumes were reduced with a SpeedVac and samples were then freeze‐dried.

Subsequently, lyophilized glycopeptides were resuspended in 200 μL of 50 mM ammonium bicarbonate (pH 8.4) and 5 μL of recombinant PNGase F (EC 3.5.1.52, Roche, Switzerland) was added to each sample for *N*‐glycan release. The reaction was carried out at 37°C, with an additional 5 μL aliquot of PNGase F added after the first 8 h, and samples were freeze‐dried after 24 h.

Samples were then resuspended in 200 μL of 5% (v/v) acetic acid and *N*‐glycans were separated from peptides and *O*‐glycopeptides via reverse phase chromatography using Sep‐Pak classic C_18_ cartridges (Waters, USA) as described previously (North et al. 2010). *N*‐glycans were collected in the 5% acetic acid fraction, whereas *O*‐glycopeptides were collected by combining the 20% and 40% (v/v) propan‐1‐ol (in 5% (v/v) acetic acid) fractions. Volumes were reduced with a SpeedVac prior to freeze‐drying.

*O*‐glycans were released by reductive elimination as follows: lyophilized *O*‐glycopeptides were resuspended in 400 μL of 0.1 M potassium hydroxide containing potassium borohydride (54 mg/mL) and incubated for 14–16 h at 45°C. The reaction was terminated by adding five drops of glacial acetic acid, followed by purification with a Dowex 1‐X8 desalting column. Volumes were reduced with a SpeedVac and freeze‐dried. Excess borates were removed via co‐evaporation with 10% (v/v) acetic acid in methanol under a stream of nitrogen gas at room temperature. *O*‐glycans were purified once more via reverse phase chromatography using Sep‐Pak classic C_18_ cartridges (Waters, USA) as described previously for the separation of *N*‐glycans from *O*‐glycopeptides (North et al. 2010). The 5% (v/v) acetic acid fraction containing *O*‐glycans was collected, its volume reduced and freeze‐dried.

Purified *N*‐ and *O*‐glycans were permethylated following the NaOH procedure and purified by reverse phase chromatography to remove hydrophilic contaminants, using Sep‐Pak classic C_18_ cartridges (Waters, USA) as described previously (North et al. 2010). The 15%, 35%, 50% and 75% (v/v) acetonitrile fractions were collected, volumes were reduced with a SpeedVac and samples were freeze‐dried.

Samples of media from DS‐1 expression (unpurified) were buffer exchanged with 1× PBS pH 7.4, reduced, denatured, digested, and permethylated as described above. The amount of protein in each of these samples was not normalised and was 400 μg for the 0.5% methanol induction condition and 72 μg for the 3.5% methanol induction condition.

Derivatized glycans were resuspended in 10 μL methanol, and 1 μL of each sample was mixed with 1 μL of DABP matrix in 75% (v/v) acetonitrile. The sample/matrix mixture was then spotted on a MALDI plate and glycans were analysed on an Applied Biosystems Sciex 4800 MALDI‐TOF/TOF mass spectrometer.

The data were analysed using Data Explorer version 4.6 from Applied Biosystems Sciex and Glycoworkbench (Ceroni et al. 2008). Structural annotations of *N*‐ and *O*‐glycans were based on monosaccharide compositions derived from the m/z value, knowledge of biosynthetic pathways and MS/MS fragmentation patterns. In the analysis of N‐ and O‐glycan relative distributions, each glycan signal intensity (monoisotopic ^12^C peak) is normalised to the total sum of glycan relative intensities for each spectrum.

### 2.9 ELISA‐Based Glycan Binding Assay

The oligosaccharide binding assay was conducted as previously described (Sun et al. 2016) with minor modifications. Purified DS‐1 (G2P[4]) VP8* proteins from methanol induction at 0.5%, 1.5%, 2.5%, and 3.5% (OD_600_ 140) were coated onto Maxisorp 96‐well plates and incubated at 4°C overnight in phosphate‐buffered saline (PBS). After blocking with 5% Bovine Serum Albumin (BSA), PAA‐biotin‐labelled mucin core 2 or Lactotetraose (LNT) glycans (CD BioGlyco) were added at a concentration of 0.4 μg/well each, and the plate was left at 4°C overnight. Following five washes with PBS containing 0.05% Tween 20, horseradish peroxidase‐conjugated streptavidin (Abcam) was added at a concentration of 0.04 μg/well. The reaction was developed using a 3,3′,5,5′‐tetramethylbenzidine kit (Thermo Fisher Scientific), and absorbance was measured at 450 nm in quadruplicate. To normalise the glycan binding capacity to protein concentration, a Histag ELISA was performed on the same plate. The binding efficiency (%) was calculated by comparing the curves derived from serial dilutions of the treatments using both assays, using the following Equation (1):

(1) $$\text{Biding Efficiency}\;\left(\%\right)=\left(\frac{\text{Glycan Binding Absorbance}}{\text{Protein Presence Absorbance}}\right)\times 100$$

### 2.10 Statistical and Graphical Analysis

Statistical analyses were conducted using RStudio (Version 2024.09.1 + 394). Spearman and Pearson correlation tests were employed to evaluate the relationship between His‐tag and RV ELISA rankings in clone screening and the methanol titration experiment, respectively. Pairwise T‐tests were performed to compare protein expression from the methanol titration experiment. Values of *p* < 0.05 were considered to indicate statistical significance. Visualisations were created using the ggplot2 package. Protein size was predicted using R software and experimentally validated via SDS‐PAGE.

## 3 Results

### 3.1 Screening of SuperMan5 Clones Expressing DS‐1 (G2P[4]) VP8* Using Indirect ELISA

The DS‐1 (G2P[4]) VP8* gene was codon optimised for *P. pastoris and* cloned in‐frame with a truncated version of the α‐mating factor secretion signal under the control of the inducible AOX1 promoter, using the pD912‐AK vector as the backbone. This truncated signal peptide has been shown to increase horseradish peroxidase expression by 137% (Lin‐Cereghino et al. 2013) and has been successfully applied in the production of the glyconjugate CRM197, achieving final purified yields exceeding 100 mg/L (Aw et al. 2021).

**FIGURE 1:**
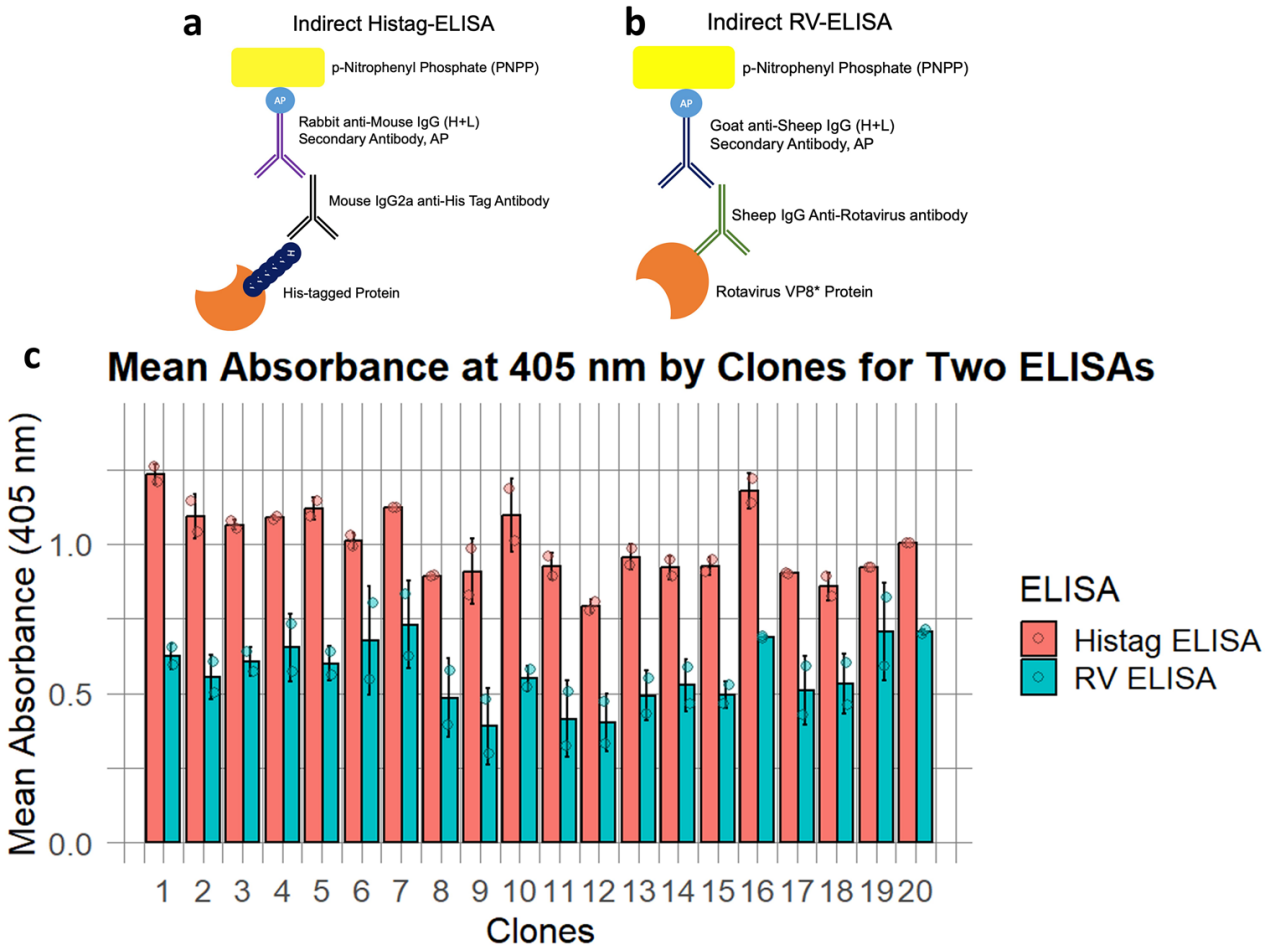
Clone screening using indirect ELISA (a) Indirect ELISA using an anti‐His tag antibody as the primary antibody (Histag‐ELISA). (b) Indirect ELISA using an anti‐rotavirus antibody as the primary antibody (RV‐ELISA). (c) Expression analysis of 20 clones secreting DS‐1 (G2P[4]) VP8* under the control of the inducible AOX1 promoter following methanol induction. Error bar indicates the standard deviation of 2 technical replicates.

To identify the highest‐secreting clones, microscale cultivation was conducted in DWP (Kaushik et al. 2020), selecting 20 clones for screening. The expression of DS‐1 (G2P[4]) VP8* was assessed using clarified supernatants through two indirect ELISAs: one utilising an anti‐His tag as the primary antibody (Histag‐ELISA) (Figure 1a) and the other employing an anti‐rotavirus antibody as the primary antibody (RV‐ELISA) (Figure 1b). ELISA offers several advantages over commonly used methods for screening secreted proteins in *P. pastoris* supernatants, such as Dot Blot (Zeder‐Lutz et al. 2006), SDS‐PAGE/western blot (Gasser et al. 2006), and Bradford assays (Karaoglan et al. 2014) with respect to sensitivity, specificity, and quantitative precision. Additionally, its compatibility with automated liquid handling facilitates large‐scale clone library screening.

Data from two ELISA assays (Figure 1c) were combined and analysed to determine the top‐secreting clones. The mean absorbance values for each clone in both assays were first normalised using min‐max normalisation to ensure comparability between the two datasets. The normalised values were then combined into a composite score by calculating the average of the normalised scores from both assays. This composite score represented the overall performance of each clone across the two assays. Equal importance was assigned to both assays in this analysis. Clones were then ranked based on their composite scores, in descending order, with higher scores indicating better candidates. The final output included the clone rank, composite score, and average standard error (Table S1). This approach balances both assay results while addressing measurement variability. The top‐ranked clone 16 was selected as the best candidate.

Additionally, a Spearman's correlation analysis was performed between the two ELISA assays, resulting in a value of 0.63, which indicates a strong correlation (Figure S1). This result demonstrates the ability of the approach to effectively distinguish between higher and lower‐secreting clones. Building on this, ELISA was employed for both screening and protein concentration measurements. During screening, the signal from each clone was adjusted by subtracting the signal of the negative control. For protein concentration measurements, a reference curve was generated using serial two‐fold dilutions of purified DS‐1 (G2P[4]) VP8* protein with a known concentration.

### 3.2 Impact of Methanol on DS‐1 (G2P[4]) VP8* Expression

**FIGURE 2:**
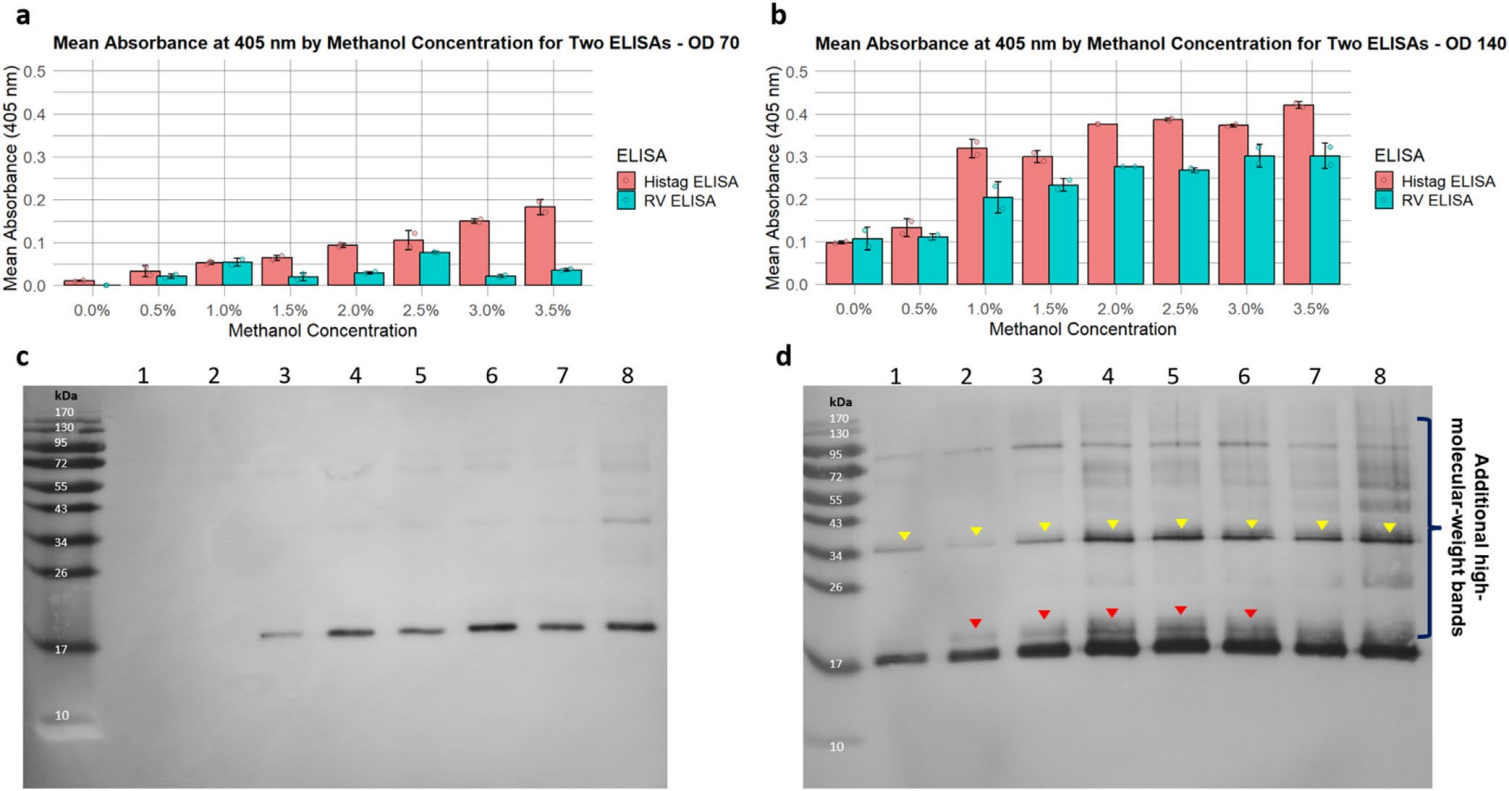
The effect of different induction conditions on protein expression. (a) Indirect ELISA using anti‐His and anti‐rotavirus antibodies to assess DS‐1 (G2P[4]) VP8* expression under eight distinct methanol induction conditions at OD_600_ 70. (b) Indirect ELISA using anti‐His and anti‐rotavirus antibodies to evaluate DS‐1 (G2P[4]) VP8* expression at OD_600_ 140. (c) Western blot corresponding to the eight methanol induction conditions at OD_600_ 70, using an anti‐His primary antibody for detection (lanes 1–8 correspond to methanol induction concentrations of 0.0%, 0.5%, 1.0%, 1.5%, 2.0%, 2.5%, 3.0%, and 3.5%). (d) Western blot analysis of the same induction conditions at OD_600_ 140, using an anti‐His primary antibody for detection(lanes 1–8 correspond to methanol induction concentrations of 0.0%, 0.5%, 1.0%, 1.5%, 2.0%, 2.5%, 3.0%, and 3.5%). Additional high‐molecular‐weight bands are observed, with a distinct band appearing well above the expected 19.2 kDa (red arrows) and a prominent band at 34 kDa (yellow arrows). For ELISAs, error bars indicate the standard deviation of two measurements, comprising two biological replicates. Since a strong Pearson correlation was observed only with samples induced at OD_600_ 140, a pairwise *t*‐test was conducted for both ELISAs at this cell density. The results are available in Tables S2 and S3. For the Western blot, the supernatants from both biological replicates were pooled, and the analysis was conducted as described in the Materials and Methods section.

To assess the potential of microscale cultivation for medium optimization, the top‐ranked clone 16 was selected for a second round of screening using a 24‐deep‐well plate to evaluate eight distinct methanol induction conditions at two different cell densities (OD_600_ 70 and 140). RV and Histag ELISAs were utilised to quantify protein expression. Comparison of both inductions revealed a significantly higher protein titre when cells were induced at OD600 140 (Figure 2). At OD_600_ 70, both ELISAs exhibited low signal (Figure 2a), aligning with the Western blot results (Figure 2b), where the expected ~19.2 kDa band remains visible, but is less pronounced. The Pearson correlation for both ELISAs (Figure S2) was low, primarily due to weak detection, especially from the RV‐ELISA. Notably, the RV‐ELISA exhibited stronger background noise, suggesting non‐specific binding to endogenous proteins in the supernatant. This makes it less suitable for screening low amounts of unpurified rotavirus proteins. In contrast, the anti‐His‐tag antibody demonstrated greater specificity, yielding more consistent results in the ELISA and Western blot comparison.

At the higher cell density (OD_600_ 140), protein expression was notably enhanced, as confirmed by both ELISAs (Figure 2c) and Western blot analysis (Figure 2d). The highest protein expression levels were achieved by inducing with BMM supplemented with 3.5% (v/v) methanol, as determined by His‐tag and RV ELISA assays. The ELISAs also demonstrated a strong Pearson correlation (*r* = 0.95) (Figure S3), showing the efficacy of combining ELISA methods for media optimization at high cell densities.

To determine whether the variation in protein expression profiles between methanol treatments was due to differences in sample concentration, we conducted an additional Western blot where protein amounts were normalised based on Histag ELISA signals (Figure S4) using samples from the condition with induction at higher cell density (OD_600_ 140). Although higher molecular weight smear bands were less prominent at higher methanol concentrations in the supplementary blot, the overall expression profile remained similar to the original blot (Figure 2D), suggesting that sample loading was not the main factor influencing the observed differences.

Thus, as evidenced by our findings, there is a clear positive correlation between increasing both cell densities and methanol concentration, resulting in higher protein titers and expression, but different protein species. This approach aligns with the widely applied high‐cell‐density fermentation (HCDF) strategy for *P. pastoris* heterologous protein expression. HCDF is commonly employed in bioreactors, leveraging the unique ability of *P. pastoris* to grow in minimal medium at high densities while secreting minimal endogenous proteins (Liu et al. 2019).

### 3.3 Impact of Methanol on DS‐1 (G2P[4]) VP8* Processing and Glycosylation

At first glance, our analysis revealed a methanol concentration‐dependent increase in protein isoforms, evidenced by the appearance of additional high‐molecular‐weight bands in the Western blot data (Figure 2b,d). Notably, a double band near the expected 19.2 kDa (red arrows) became more distinct, with the upper band intensifying with induction at OD_600_ 140 (Figure 2d). This methanol‐dependent increase in protein species raised questions about the efficiency of post‐translational processing under different conditions.

**FIGURE 3:**
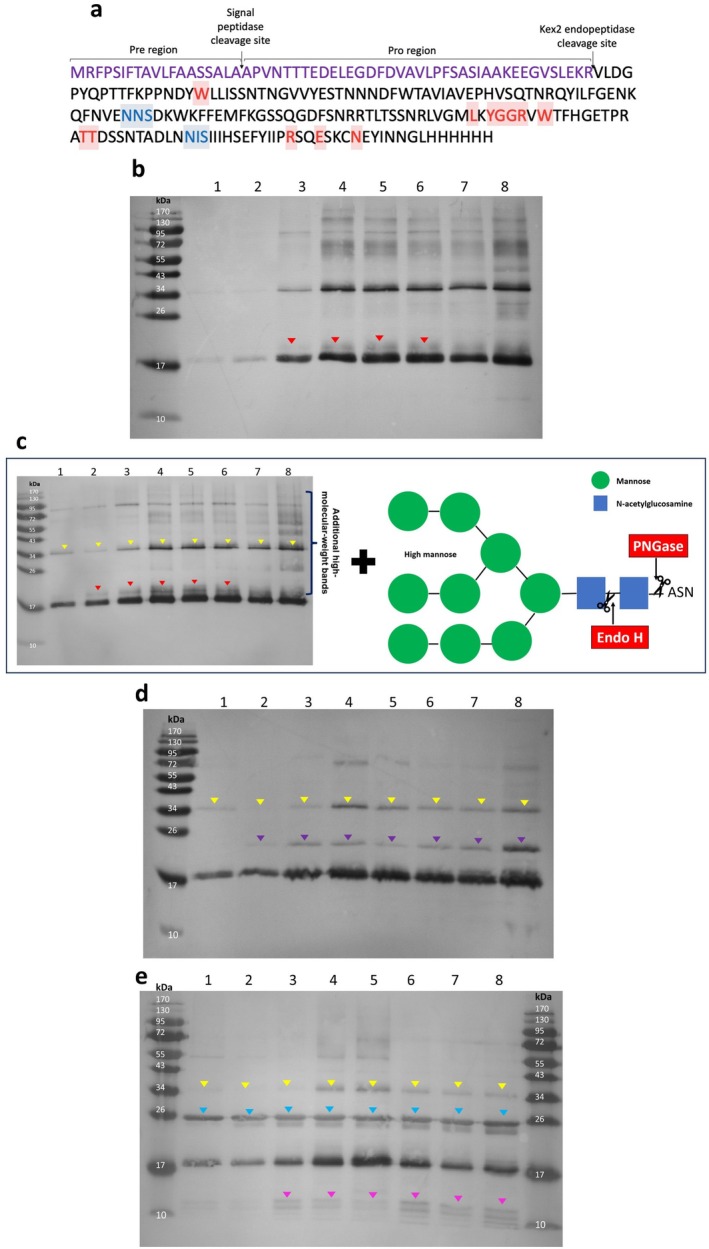
Enzymatic treatment to elucidate the identity of higher molecular weight bands (a) Protein sequence highlighting key features: The signal peptide with cleavage sites (purple), the truncated DS‐1 (G2P[4]) VP8* sequence (black), glycan‐binding cavity residues (red) as identified by (Sun et al. 2020), and potential *N*‐linked glycosylation motifs (blue) predicted using the NetNGlyc tool (Steentoft et al. 2013). (b) Western blot analysis of Kex2 digestion across eight methanol induction conditions at OD_600_ 140, with lanes 1–8 corresponding to methanol concentrations of 0.0%, 0.5%, 1.0%, 1.5%, 2.0%, 2.5%, 3.0%, and 3.5%. A distinct band remains well above the expected 19.2 kDa (red arrows) even after Kex2 cleavage. (c) Schematic representation of Peptide: N‐glycosidase F (PNGase F) and Endoglycosidase H (Endo H) cleavage sites within a high‐mannose glycan structure. (d) Western blot analysis of PNGase F‐mediated deglycosylation under the same methanol induction conditions (lanes 1–8 as described above), 34 kDa bands observed in untreated supernatants became less prominent (yellow arrows), while new bands emerged around 22 kDa (purple arrows). (e) Western blot analysis of Endo H–mediated deglycosylation under identical methanol induction conditions (lanes 1–8, as described above). Prominent bands at ~34 kDa (yellow arrows) were reduced in intensity following Endo H treatment, accompanied by the appearance of a doublet at ~26 kDa (blue arrows) and additional lower‐molecular‐weight species at ~10 kDa (pink arrows). For all Western blots, supernatants from biological replicates were pooled, and the analysis was performed as detailed in the Materials and Methods section.

The secretion of recombinant proteins via the truncated α‐mating factor signal occurs in two steps: (i) removal of the pre‐signal by signal peptidases in the endoplasmic reticulum (ER) and (ii) cleavage of the pro‐leader sequence by Kex2 endopeptidase in the Golgi apparatus (Figure 3a) (Lin‐Cereghino et al. 2013). High methanol concentrations may drive excessive recombinant protein production, causing ER stress and overloading the Golgi, thereby impairing proper processing and glycosylation. If Golgi processing is incomplete, the molecular weight of the protein would correspond to 23.2 kDa, which could explain the band observed directly above the expected 19.2 kDa. To investigate this, we treated the supernatants from the inductions at OD_600_ 140 with Kex2 enzyme. However, while the upper band became less prominent, it remained detectable (Figure 3b—red arrows), suggesting that the majority of the signal peptide is correctly processed. Furthermore, the similarity between the Western blot profiles of Kex2‐treated (Figure 3b) and untreated samples (Figure 2d) further supports the proper processing of the signal peptide.

Although unexpected given that our host was the *P. pastoris* SuperMan5 strain, we investigated whether these bands resulted from differential *N*‐linked glycosylation, as this has already been reported by (Apte‐Deshpande et al. 2009) who found that proteins produced by *P. pastoris* can be secreted in both glycosylated and non‐glycosylated forms. To test this, supernatants from inductions at OD_600_ 140 were treated with Peptide: N‐glycosidase F (PNGase F) (Figure 3c), leading to noticeable shifts in gel band patterns. The high‐molecular‐weight smear bands disappeared, the 34 kDa bands observed in untreated supernatants became less prominent (yellow arrows—Figure 3d), and new bands emerged around 22 kDa (purple arrows Figure 3d lanes 2–8). Additionally, the double band near the target size disappeared, further supporting the role of N‐glycosylation in these modifications (Figure 3d). Noticeable shifts in the gel profile were also observed following Endo H treatment, including the weakening of the 34 kDa bands, the appearance of a doublet at ~26 kDa (blue arrows) and additional lower‐molecular‐weight species at ~10 kDa (pink arrows) (Figure 3e). Interestingly, certain higher‐molecular‐weight species persisted after both deglycosylation treatments, suggesting the presence of O‐glycosylation and/or incomplete removal of N‐glycans. Such incomplete deglycosylation can occur when proteins possess multiple closely spaced glycosylation sites with bulky glycans, where steric hindrance limits PNGase F and Endo H accessibility, leaving some glycans attached, particularly within densely glycosylated regions.

The appearance of lower molecular weight bands likely reflects increased susceptibility to proteolytic cleavage following N‐glycan removal. This fragmentation pattern may be further influenced by endogenous proteases, as the vacuolar enzymes Prb1 and Pep4 could leak into the supernatant, while Yps1—a cell‐surface or secreted aspartyl protease—may contribute directly to extracellular degradation. To verify that the production strain was *P. pastoris* GlycoSwitch SuperMan5‐10 GS 10010 Genotype: och1‐∆1, GAP‐mannosidaseHDEL, and to determine the target gene copy number, whole‐genome sequencing was performed using nanopore technology (Full Circle Labs Ltd) (Figure S5). The analysis confirmed the insertion of the *Trichoderma reesei* α‐mannosidase gene at the OCH1 locus, with no mutations, validating that the correct strain was used. The genome analysis also confirmed a single‐copy integration of the DS‐1 (G2P[4]) VP8* gene at the AOX1 locus.

In order to assess whether the observed increase in glycosylation was protein‐dependent, we expressed another rotavirus variant, WA (G1P[8]) VP8* (Figure S6a), under two methanol concentrations: 0.5% and 3.5%. A similar trend was observed, with higher protein expression at the higher methanol concentration (Figure S6b), accompanied by an increase in higher molecular weight glycoforms, as indicated by the appearance of additional high–molecular weight bands in the Western blot (Figure S6c).

### 3.4 *N*‐ and *O*‐Glycomic Profiling of DS‐1 (G2P[4]) VP8

**FIGURE 4:**
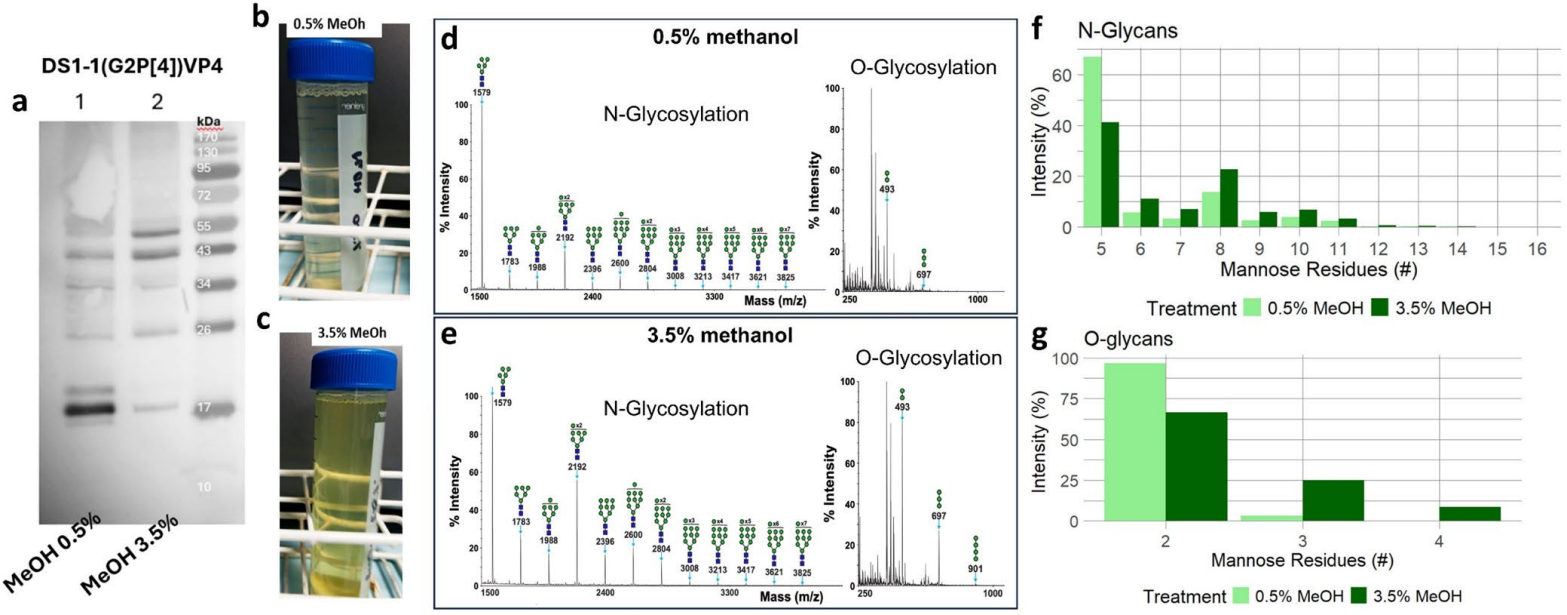
Comparison of glycosylation patterns of proteins expressed using two different methanol induction conditions (a) Western blot analysis of purified DS‐1 (G2P[4]) VP8* produced under two different methanol induction conditions (0.5%, lane 1% and 3.5%, lane 2) using baffled shake flasks (samples were normalized, and 300 ng was loaded into each well). (b and c) Clarified supernatants from both induction conditions. (d) MALDI‐TOF MS profile of permethylated N‐ and O‐linked glycans from SuperMan5 *P. pastoris*‐produced DS‐1 (G2P[4]) VP8 under 0.5% methanol induction. (e) Corresponding profile from the same protein expressed at 3.5% methanol. N‐glycans were obtained from the 50% MeCN fraction, and O‐glycans from the 35% MeCN fraction. All molecular ions represent the singly charged and sodiated form [M + Na]^+^. Structures shown with brackets have not had their antennal location unequivocally defined. Additional *N*‐ and *O*‐glycan MALDI‐TOF MS profiles from different solvent conditions are available in (Figures S7–S10). (f and g) Relative distribution of *N‐/O‐glycan* structures. The bar heights indicate the relative abundance of larger glycan structures compared to (f) Man5 and (g) dimeric mannose (Man2). Glycomics measurements were conducted as single determinations (*n* = 1).

To more precisely investigate the types of glycosylation and examine the glycan profiles of the target protein, we conducted *N*‐ and *O*‐glycomic analyses on purified DS‐1 (G2P[4]) VP8*. To enhance protein yield and obtain two distinct sample types, we replicated two conditions from the methanol titration screening using baffled shake flasks. Protein expression was induced at an OD_600_ of 140 using 0.5% and 3.5% methanol, followed by Histag purification. Consistent with the microscale culture, the 0.5% methanol condition produced a prominent band at 19.2 kDa, accompanied by faint higher molecular weight bands, whereas the 3.5% methanol condition led to the formation of additional multiple stronger and higher molecular weight species (Figure 4a). Notably, the supernatant from the 3.5% induction exhibited a pronounced yellow coloration compared to the 0.5% condition (Figure 4b), suggesting the presence of pigment impurities. These impurities, likely flavins, have been reported by Surribas et al. (2007) and Marx et al. (2008) and, as highlighted by Kongsinkaew et al. (2023), can interfere with the separation and purification of recombinant proteins. The final yield of purified protein from the culture supernatant was 0.5 mg/L under the 0.5% methanol condition and 10 mg/L under the 3.5% methanol condition.

*N*‐Glycomics analysis revealed that the mannose‐5 (Man5) glycan exhibited the highest peak intensity in both purified DS‐1 (G2P[4]) VP8* samples (Figure 4d,e). While larger high‐mannose glycans were also present, their abundance was notably greater in the protein expressed with induction using 3.5% methanol, as reflected by the peak intensities in Figure 4. The results also show a noticeable decrease in the proportion of Man5 when expression is induced using 3.5% methanol with a shift towards higher mannose structures (Figure 4f). A similar pattern is observed in MALDI‐TOF MS spectra of *N*‐glycans from the 75% MeCN fraction, where the abundance of larger high‐mannose structures increased with methanol concentration, and trace amounts of *N*‐glycans with up to 19 mannoses were detected in the protein expressed with 0.5% methanol (Figure S7a) and up to 26 mannoses in the 3.5% methanol condition (Figure S7b). This variation highlights a strong correlation between methanol concentration and glycan size, where higher methanol levels promote processing to *N*‐glycans with a greater number of mannose residues.

Regarding *O*‐glycosylation, Figure 4d,e,g also shows a shift towards higher mannose addition when expression is induced using 3.5% methanol compared to 0.5% methanol. When protein expression is induced with 0.5% methanol, the predominant *O*‐linked glycan detected is dimeric, while for 3.5% substantial amounts of trimeric and detectable amounts of tetrameric structures are detected, aligning with previous observations (Duman‐Özdamar and Binay 2021; Gomathinayagam and Hamilton 2014; Radoman et al. 2021). However, larger *O*‐glycan structures were also detected, reaching up to 11 mannose residues in the purified sample from cultures induced with 0.5% methanol and extending to 15 mannoses in the sample purified from cultures induced with 3.5% methanol in the 75% MeCN fraction, (Figure S9; Duman‐Özdamar and Binay 2021; Gomathinayagam and Hamilton 2014; Radoman et al. 2021). Elevated methanol concentrations also resulted in a greater abundance of larger high‐mannose structures in non‐purified supernatants, with samples induced at 3.5% methanol exhibiting more of these structures than those at 0.5%. This highlights the impact of methanol on both the target protein and the overall glycosylation profile of the *Pichia* secretome, including *N*‐ and *O*‐glycosylation (Figures S8 and S10).

We also examined whether methanol concentration could influence the expression of α‐mannosidases, potentially preventing *T. reesei* α‐1,2‐mannosidase from converting Man₈GlcNAc₂ to Man₅GlcNAc₂, which would explain the increased abundance of larger N‐linked glycans at elevated methanol concentrations. However, no significant differences in α‐mannosidase activity were detected in cell lysates from cultures grown at 0.0%, 0.5%, or 3.5% methanol. Furthermore, Western blot analysis using an anti‐HDEL (ER‐retention tag present in the *T. reesei* α‐1,2‐mannosidase) antibody revealed no detectable differences in protein expression between lysate samples from the same methanol treatments (Figure S11a,b). Based on these results, we hypothesize that, since enzyme activity remains unchanged, the cellular capacity for N‐linked glycan trimming may become insufficient to process the increased load resulting from the elevated protein expression observed at higher methanol concentrations.

### 3.5 DS‐1 (G2P[4]) VP8 Glycoprotein: Functional Assay

**FIGURE 5:**
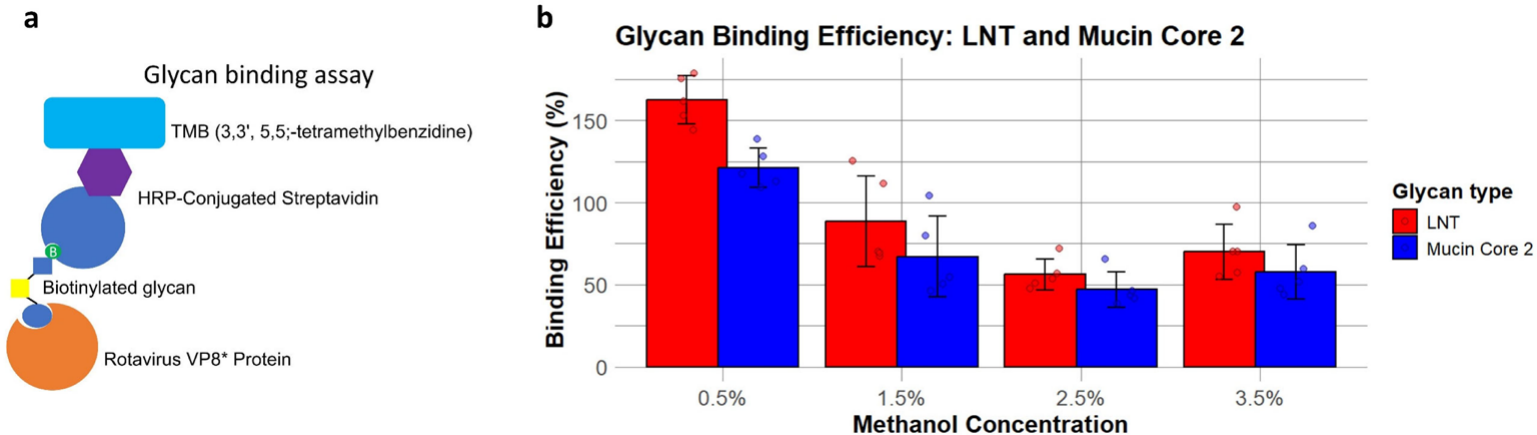
Assessing glycan binding efficiency as a measure of functionality. (a) Glycan ELISA assay to assess protein‐glycan interactions. (b) Glycan binding efficiency of purified DS‐1 (G2P[4]) VP8* expressed under methanol induction conditions of 0.5%, 1.5%, 2.5%, and 3.5% (OD600 140).

Rotaviruses exploit host glycans as receptors for cell attachment (Ramani et al. 2016). Thus, the ability of the recombinant VP8 to bind glycans can serve as an indicator of whether the protein retains the necessary structure needed to act as a good antigen. To assess the impact of glycosylation on protein functionality, we evaluated the glycan‐binding properties of DS‐1 (G2P[4]) VP8* purified from *P. pastoris* supernatants under four different methanol induction conditions: 0.5%, 1.5%, 2.5%, and 3.5% (induced at OD_600_ 140). As shown in Figure 5, the glycan‐binding efficiency of the less glycosylated samples from the 0.5% methanol condition was significantly higher, approximately two‐fold greater.

Notably, DS‐1 (G2P[4]) VP8* expressed in *P. pastoris* exhibited binding to both mucin core 2 and LNT glycans, in contrast to the same protein produced in *E. coli* , which could bind only to mucin core 2 (Liu et al. 2016; Sun et al. 2020). One possible explanation is that glycosylation, to some extent, may enhance the ability of a protein to bind to glycans by stabilizing its structure, thereby facilitating crucial carbohydrate–carbohydrate and carbohydrate–protein interactions involved in molecular recognition, cell adhesion, and immune responses (Quintana et al. 2023). However, the presence of larger glycan structures may mask the binding sites or contribute to steric hindrance.

## 4 Discussion

An unexpected finding in our study was the pronounced heterogeneity in the *N*‐glycosylation of our target protein. Although (Laukens et al. 2020) reported two predominant mannose structures, Man_5_GlcNAc_2_ and Man_9_GlcNAc_2_, with minor signals corresponding to Man_8_GlcNAc_2_ and Man_10_GlcNAc_2_ when expressing mouse interleukin‐22 in *P. pastoris* SuperMan5, this strain was specifically engineered to yield a more homogeneous *N*‐glycan profile, primarily featuring Man_5_GlcNAc_2_. The SuperMan5 strain was developed through a single genetic modification involving the disruption of *OCH1*, to prevent hypermannosylation, combined with the integration of an α‐1,2‐mannosidase from *T. reesei*—tagged with an HDEL signal for ER‐Golgi localization—at the same locus. This enzyme is expressed under the control of the constitutive GAP promoter and should trim Man_8_GlcNAc_2_ to Man_5_GlcNAc_2_, thereby reducing glycan heterogeneity (Gehlsen and Chappell 2017; Jacobs et al. 2009). However, our data indicate that under some conditions, the processing is impaired or inefficient. Several possible explanations exist, including the ratio of enzyme to heterologous protein, or the existence of alternative enzymes/metabolic pathways for mannose addition that are not affected by the *OCH1 knockout and that have higher activity under some bioprocessing conditions*.

In previous work, comparative genomics and transcriptomics of *P. pastoris* identified over 30 genes associated with *N*‐ and *O*‐glycosylation, across *K. pastoris* wild‐type, *K. phaffii* wild‐type, and *K. phaffii* GS115 strains (Love et al. 2016). Genes involved in N‐glycosylation—including the ALG family (*ALG1, ALG2, ALG3, ALG9, ALG11, ALG12*), *DPM1, MNN2, KTR4*, and *GPI18*—as well as O‐glycosylation‐related genes such as the *PMT* family and *BMT1* (implicated in the beta‐mannosylation of outer chains of glycans), were identified in their genome and also confirmed to be present and conserved in the SuperMan5 strain used in the present study (Table S4). Apart from the *ALG* gene family, which mediates the stepwise addition of sugars to assemble the full‐length N‐glycan precursor prior to its transfer to proteins and does not play a role in additional mannosylation (Gao et al. 2004), these genes may function as a compensatory mechanism, maintaining mannose incorporation. Consistent with this, (Hopkins et al. 2011) showed that targeted knockouts of *BMT2*, *BMT3*, and *BMT1* effectively eliminated β‐mannose‐linked glycans and their associated antigenicity in recombinant human erythropoietin (rhEPO), produced in a *P. pastoris* strain previously engineered to secrete human glycoproteins bearing fully complex, terminally sialylated N‐glycans (Hamilton et al. 2006).

Our results indicate that the expression of the α‐1,2‐mannosidase under the control of the *PGAP* promoter is largely unaffected by methanol concentration in the growth medium. Specifically, enzyme activity assays showed no significant differences in α‐mannosidase activity in cell lysates from cultures grown at 0.0%, 0.5%, or 3.5% methanol. Complementary western blot analysis using an anti‐HDEL antibody corroborated these findings, revealing consistent protein levels across the same methanol treatments.

Notably, (Love et al. 2016) reported a strong correlation in gene expression between OD_600_ 2 and OD_600_ 20, indicating that genes were consistently up‐ or downregulated regardless of cell density. Furthermore, genes involved in protein glycosylation were expressed at low levels when strains were cultured in methanol, while GAP expression remained stable across different carbon sources (Figure S11).

In contrast to these findings, (Waterham et al. 1997) suggested that GAP‐driven expression could be reduced under methanol conditions, as reflected in the diminished GAPDH activity in methanol‐grown cultures and the reduced intracellular β‐lactamase activity in the *PGAP‐bla* construct compared to growth on glucose or glycerol. Importantly, whether GAP promoter activity is maintained or partially attenuated, conditions that drive higher protein production may overwhelm the folding and glycosylation machinery, thereby reducing processing efficiency. Such saturation would be consistent with the pronounced glycoform heterogeneity observed under elevated methanol concentrations. In particular, the cellular capacity for glycan trimming—such as the activity of 1,2‐α‐mannosidase—may become insufficient to process the excessive glycan load presented by the elevated protein flux. Importantly, it is a fact that our study employed significantly higher cell densities, an engineered strain carrying two resistance cassettes, and elevated methanol concentrations, all of which might influence gene expression profiles. These factors may contribute to the observed glycosylation heterogeneity by altering the regulation of glycosylation‐related genes or metabolic pathways. Further investigations, including transcriptomic and proteomic analyses under these specific conditions, could provide deeper insights into the underlying mechanisms driving this unexpected glycosylation complexity.

Regarding protein functionality, another possible approach is to produce high glycoprotein titers, purify the product, and then carry out in vitro deglycosylation. This approach was employed by (Idrovo‐Hidalgo et al. 2024), who deglycosylated the SARS‐CoV‐2 Spike Receptor Binding Domain produced in *P. pastoris* and compared the glycosylated and deglycosylated forms. Their findings align closely with ours: the deglycosylated variant, when used as an immunogen, elicited a humoral immune response ten times stronger than that of the glycosylated form, with enhanced neutralising antibody activity and a markedly more robust cellular immune response. Although this strategy is effective, it introduces an additional enzymatic step, which can significantly increase the overall cost of the final product. Therefore, controlling the bioprocess to obtain the desired isoform—or to increase its proportion—is essential for a more cost‐effective scale‐up.

Most research on controlling protein glycosylation during bioprocessing has focused on antibody production in mammalian cells. These studies have demonstrated that fermentation parameters—such as pH, temperature, dissolved oxygen, culture mode, and harvest time—as well as media supplementation with carbon and nitrogen sources or glycan precursors, can effectively modulate mAb glycosylation patterns (Edwards et al. 2022; Puranik et al. 2022). In our study, we showed that cell density and methanol concentration are key variables that can be strategically leveraged to optimise glycoprotein production, highlighting the potential of the *P. pastoris* secretion system as a platform for protein glycosylation control.

## 5 Conclusions

In conclusion, this research underscores the feasibility of an ELISA‐based screening system for *P. pastoris* clone selection and media optimization, offering seamless adaptability to liquid handling and automation. It also underscores the crucial role of SDS‐PAGE and Western blot in evaluating protein integrity and detecting variants, such as glycoforms, which can significantly impact functionality. Notably, glycan‐binding analysis of DS‐1 (G2P[4]) VP8* revealed that less glycosylated samples exhibited nearly twice the binding efficiency. Furthermore, the findings reveal that methanol concentration influences glycosylation complexity, not only affecting the target protein but also shaping the broader *Pichia* secretome. We emphasize the importance of fine‐tuning cultivation parameters to enhance protein yield, optimize glycosylation profiles, and minimize supernatant impurities—critical factors for both scale‐up and downstream processing. By streamlining bioprocess optimization, this approach accelerates production while reducing strain burden and the need for extensive genetic modifications.

Leveraging these insights and our comprehensive data, future work to develop a deeper understanding of the protein glycosylation machinery in bioprocessing requires a multi‐omics approach. Such analyses, ideally conducted in high‐density cultures within a controlled bioreactor environment, could provide critical insights into cellular responses, glycosylation‐associated pathways, metabolic shifts, and secretion dynamics. Furthermore, we believe that the use of Design of Experiments (DoE)‐based strategies to systematically optimize cultivation conditions, enabling precise control over protein expression and ensuring that glycosylation—whether retained or minimized—aligns with the intended application. Overall, the link between bioprocessing conditions and glycosylation could offer another lever to fine‐tune protein quality to maximize functionality.

## Author Contributions

A.O. developed methods, designed and performed experiments, analysed data and wrote the paper. Z.P. and X.C. performed experiments. R.D. designed, conducted, and analysed Glycomics experiments and contributed to writing the paper. S.H. designed and analysed Glycomics experiments. K.P. conceived the project, designed the experiments, and contributed to writing the paper.

## Funding

This work was supported by the Department of Health and Social Care and the Engineering and Physical Sciences Research Council (EP/Y530529/1).

## Conflicts of Interest

The authors declare no conflicts of interest.

## Supporting information


**Appendix S1:** mbt270272‐sup‐0001‐AppendixS1.docx.

## Data Availability

All data are contained within the manuscript and the Appendix S1.
