# Supplementary material for: The Impact of Methanol Concentration on Recombinant Protein Glycosylation in Pichia pastoris SuperMan5
Source: Microb Biotechnol. 2025 Dec 5;18(12):e70272. doi: 10.1111/1751-7915.70272 (PMC12679317; doi:10.1111/1751-7915.70272)
Supplement: Supplementary file 1 — Appendix S1: mbt270272‐sup‐0001‐AppendixS1.docx. [file MBT2-18-e70272-s001.docx]

**Supplementary Material**

**Cloning Strategy and strain construction**

The DS-1 (G2P[4]) VP8* fragment was amplified via PCR using the primers DS_1_Fwr and DS_1_H6_Rev to incorporate a 6× HIS tag. The reaction was carried out with Phusion DNA Polymerase (ThermoFisher Scientific) following the manufacturer’s protocol. The annealing temperature was calculated using the ThermoFisher online Tm calculator (https://www.thermofisher.com/tmcalculator.html), and the extension time was set to 15 seconds. PCR fragment was purified from agarose gels using the Zymoclean™ Gel DNA Recovery Kit (Zymo Research Corporation, Irvine, USA) and served as template for a second PCR with primers DS_1_G2P4_VP4_GA_Fwd and DS_1_G2P4_VP4_GA_Rev to add homology assembly overhangs. The PD912-AK vector (ATUM, Newark, USA) was linearized by double digestion with XhoI and AfeI, and both the digested vector and the second PCR product were purified using the Zymoclean™ Clean and Concentrator Kit (Zymo Research Corporation). The vector and insert were assembled using a custom Gibson assembly master mix, prepared following the protocol available at Protocols.io ([dx.doi.org/10.17504/protocols.io.n9xdh7n](https://dx.doi.org/10.17504/protocols.io.n9xdh7n)). All DNA sequences are provided bellow:

>DS-1 (G2P[4]) VP8_ gene_art

GTTTTGGATGGTCCATACCAGCCAACTACCTTCAAGCCACCAAACGACTACTGGTTGTTGATCTCCTCCAACACCAACGGTGTTGTTTACGAGTCCACCAACAACAACGATTTCTGGACTGCTGTTATCGCCGTTGAACCACACGTTTCCCAAACTAACAGACAGTACATCCTGTTCGGTGAGAACAAGCAGTTCAACGTCGAAAACAACTCCGACAAGTGGAAGTTCTTCGAGATGTTCAAGGGTTCTTCCCAGGGTGACTTCTCCAACAGAAGAACTTTGACCTCTTCCAACAGACTGGTCGGTATGTTGAAGTACGGTGGTAGAGTTTGGACTTTCCACGGTGAAACTCCAAGAGCTACTACCGACTCTTCTAACACTGCCGACTTGAACAACATCTCCATCATCATCCACTCCGAGTTCTACATCATCCCAAGATCTCAAGAGTCCAAGTGCAACGAGTACATCAACAACGGTTTG

>DS_1_Fwr

TCGAGAAAAGAGTTTTGGATGGTCCATACCAGCC

>DS_1_H6_Rev

TTAATGATGATGATGATGATGCAAACCGTTGTTGATGTAC

> DS_1_G2P4_VP4_GA_Fwd

TAAAGAAGAAGGGGTATCTCTCGAGAAAAGAGTTTTGG

>DS_1_G2P4_VP4_GA_Rev

CGCCCCTTAACCTGAAGAGCTTAATGATGATGATGATGATGC

An identical cloning strategy was employed for the construction of the strain expressing WA (G1P[8]) VP8. The gene sequence and primers are listed below:

> WA (G1P[8]) VP8_ gene_art

TTGGATGGTCCATACCAGCCAACTACTTTCACTCCACCAAACGACTACTGGATCCTGATCAACTCCAACACCAACGGTGTTGTTTACGAGTCCACTAACAACTCCGATTTCTGGACTGCTGTTGTTGCTATCGAGCCACACGTTAACCCAGTTGACAGACAGTACACTATCTTCGGTGAGTCCAAGCAGTTCAACGTCAGAAACGACTCCAACAAGTGGAAGTTCTTGGAGATGTTCAGATCCTCCAGCCAGAACGAGTTCTACAACAGAAGAACTTTGACCTCCGACACCAGATTGGTCGGTATCTTGAAGTACGGTGGTAGAGTTTGGACTTTCCACGGTGAAACTCCAAGAGCTACTACTGACTCTTCCTCCACTGCCAACTTGAACAACATTTCCATCACCATCCACTCCGAATTCTACATCATCCCAAGATCTCAAGAGTCCAAGTGCAACGAGTACATCAACAACGGTTTG

>Wa_VP8_Fwr

TCGAGAAAAGATTGGATGGTCCATACCAGCC

>Wa_VP8_Rev

TTAATGATGATGATGATGATGCAAACCGTTGTTGATGTACTCG

>Wa_VP8_GA_Fwr

TAAAGAAGAAGGGGTATCTCTCGAGAAAAGATTGGATG

>Wa_VP8_GA_Rev

CGCCCCTTAACCTGAAGAGCTTAATGATGATGATGATGATGC

The assembled product was transformed into *E. coli* DH5α, and the plasmid was linearized at a unique SacI restriction site within the AOX1 promoter. For cloning into P. pastoris, 10 µg of the linearized plasmid DNA was introduced into the *P. pastoris* GlycoSwitch® SuperMan5 strain via electroporation, following the EasySelect™ Pichia Expression Kit protocol. Cultures were incubated for 3 days at 30 °C on YPDS medium (1% yeast extract, 2% peptone, 2% dextrose, 1% sorbitol, 2% agar) supplemented with 500 µg/mL Zeocin (Thermo Fisher Scientific). Individual colonies were subsequently streaked onto YPD plates containing the same concentration of Zeocin and incubated for 3 days to prepare for liquid culture screening.

**Table 1. Clone Performance Composite Score Table**

| **Clones** | **composite_score** | **avg_se** | **conditions** | **rank** |
| --- | --- | --- | --- | --- |
| 16 | 0.875977789 | 0.02271257 | 2 | 1 |
| 7 | 0.874266672 | 0.052125 | 2 | 2 |
| 1 | 0.844624448 | 0.02686796 | 2 | 3 |
| 4 | 0.723868186 | 0.04264215 | 2 | 4 |
| 20 | 0.70374627 | 0.003875 | 2 | 5 |
| 5 | 0.682225153 | 0.03310785 | 2 | 6 |
| 6 | 0.670195428 | 0.07399349 | 2 | 7 |
| 3 | 0.628134104 | 0.02286174 | 2 | 8 |
| 19 | 0.611610348 | 0.058125 | 2 | 9 |
| 2 | 0.58297262 | 0.05323338 | 2 | 10 |
| 10 | 0.580803194 | 0.05814841 | 2 | 11 |
| 14 | 0.34753463 | 0.04474978 | 2 | 12 |
| 13 | 0.338371961 | 0.04418897 | 2 | 13 |
| 15 | 0.308974972 | 0.02619235 | 2 | 14 |
| 17 | 0.304511659 | 0.04191532 | 2 | 15 |
| 18 | 0.284595426 | 0.05138873 | 2 | 16 |
| 8 | 0.256288176 | 0.046375 | 2 | 17 |
| 11 | 0.188985498 | 0.06199978 | 2 | 18 |
| 9 | 0.130706921 | 0.08439845 | 2 | 19 |
| 12 | 0.019145803 | 0.04260116 | 2 | 20 |

**Table 2. Pairwise T-Test Analysis of Methanol Concentration Treatments using the His-Tag ELISA (OD 140)**

| **Comparison** | **Mean_1** | **Mean_2** | **P_Value** |
| --- | --- | --- | --- |
| MeOH 0.0% vs MeOH 0.5% | 0.09775 | 0.13275 | 0.14044082 |
| MeOH 0.0% vs MeOH 1.0% | 0.09775 | 0.31875 | 0.0050091 |
| MeOH 0.0% vs MeOH 1.5% | 0.09775 | 0.29925 | 0.00260662 |
| MeOH 0.0% vs MeOH 2.0% | 0.09775 | 0.37625 | 8.06E-05 |
| MeOH 0.0% vs MeOH 2.5% | 0.09775 | 0.38675 | 0.00014963 |
| MeOH 0.0% vs MeOH 3.0% | 0.09775 | 0.37275 | 0.00016525 |
| MeOH 0.0% vs MeOH 3.5% | 0.09775 | 0.42075 | 0.00034967 |
| MeOH 0.5% vs MeOH 1.0% | 0.13275 | 0.31875 | 0.01277279 |
| MeOH 0.5% vs MeOH 1.5% | 0.13275 | 0.29925 | 0.01100694 |
| MeOH 0.5% vs MeOH 2.0% | 0.13275 | 0.37625 | 0.00352724 |
| MeOH 0.5% vs MeOH 2.5% | 0.13275 | 0.38675 | 0.00333896 |
| MeOH 0.5% vs MeOH 3.0% | 0.13275 | 0.37275 | 0.00373762 |
| MeOH 0.5% vs MeOH 3.5% | 0.13275 | 0.42075 | 0.002887 |
| MeOH 1.0% vs MeOH 1.5% | 0.31875 | 0.29925 | 0.40127404 |
| MeOH 1.0% vs MeOH 2.0% | 0.31875 | 0.37625 | 0.06559635 |
| MeOH 1.0% vs MeOH 2.5% | 0.31875 | 0.38675 | 0.04939257 |
| MeOH 1.0% vs MeOH 3.0% | 0.31875 | 0.37275 | 0.07513092 |
| MeOH 1.0% vs MeOH 3.5% | 0.31875 | 0.42075 | 0.02502767 |
| MeOH 1.5% vs MeOH 2.0% | 0.29925 | 0.37625 | 0.0164512 |
| MeOH 1.5% vs MeOH 2.5% | 0.29925 | 0.38675 | 0.01359519 |
| MeOH 1.5% vs MeOH 3.0% | 0.29925 | 0.37275 | 0.01910589 |
| MeOH 1.5% vs MeOH 3.5% | 0.29925 | 0.42075 | 0.0087081 |
| MeOH 2.0% vs MeOH 2.5% | 0.37625 | 0.38675 | 0.05228327 |
| MeOH 2.0% vs MeOH 3.0% | 0.37625 | 0.37275 | 0.29647353 |
| MeOH 2.0% vs MeOH 3.5% | 0.37625 | 0.42075 | 0.0149345 |
| MeOH 2.5% vs MeOH 3.0% | 0.38675 | 0.37275 | 0.05825809 |
| MeOH 2.5% vs MeOH 3.5% | 0.38675 | 0.42075 | 0.03015356 |
| MeOH 3.0% vs MeOH 3.5% | 0.37275 | 0.42075 | 0.01547523 |

**Table 3. Pairwise T-Test Analysis of Methanol Concentration Treatments using the RV ELISA**

**(OD 140)**

| **Comparison** | **Mean_1** | **Mean_2** | **P_Value** |
| --- | --- | --- | --- |
| MeOH 0.0% vs MeOH 0.5% | 0.107 | 0.111 | 0.8575059 |
| MeOH 0.0% vs MeOH 1.0% | 0.107 | 0.204 | 0.09480103 |
| MeOH 0.0% vs MeOH 1.5% | 0.107 | 0.233 | 0.02904411 |
| MeOH 0.0% vs MeOH 2.0% | 0.107 | 0.276 | 0.01240491 |
| MeOH 0.0% vs MeOH 2.5% | 0.107 | 0.268 | 0.01423439 |
| MeOH 0.0% vs MeOH 3.0% | 0.107 | 0.301 | 0.01864881 |
| MeOH 0.0% vs MeOH 3.5% | 0.107 | 0.301 | 0.02065157 |
| MeOH 0.5% vs MeOH 1.0% | 0.111 | 0.204 | 0.07236247 |
| MeOH 0.5% vs MeOH 1.5% | 0.111 | 0.233 | 0.00966718 |
| MeOH 0.5% vs MeOH 2.0% | 0.111 | 0.276 | 0.00091701 |
| MeOH 0.5% vs MeOH 2.5% | 0.111 | 0.268 | 0.00165921 |
| MeOH 0.5% vs MeOH 3.0% | 0.111 | 0.301 | 0.01052403 |
| MeOH 0.5% vs MeOH 3.5% | 0.111 | 0.301 | 0.0126639 |
| MeOH 1.0% vs MeOH 1.5% | 0.204 | 0.233 | 0.41230971 |
| MeOH 1.0% vs MeOH 2.0% | 0.204 | 0.276 | 0.10941275 |
| MeOH 1.0% vs MeOH 2.5% | 0.204 | 0.268 | 0.13545084 |
| MeOH 1.0% vs MeOH 3.0% | 0.204 | 0.301 | 0.09480103 |
| MeOH 1.0% vs MeOH 3.5% | 0.204 | 0.301 | 0.10104224 |
| MeOH 1.5% vs MeOH 2.0% | 0.233 | 0.276 | 0.05964586 |
| MeOH 1.5% vs MeOH 2.5% | 0.233 | 0.268 | 0.0960025 |
| MeOH 1.5% vs MeOH 3.0% | 0.233 | 0.301 | 0.09033662 |
| MeOH 1.5% vs MeOH 3.5% | 0.233 | 0.301 | 0.10308656 |
| MeOH 2.0% vs MeOH 2.5% | 0.276 | 0.268 | 0.18350342 |
| MeOH 2.0% vs MeOH 3.0% | 0.276 | 0.301 | 0.3188289 |
| MeOH 2.0% vs MeOH 3.5% | 0.276 | 0.301 | 0.35600368 |
| MeOH 2.5% vs MeOH 3.0% | 0.268 | 0.301 | 0.23130993 |
| MeOH 2.5% vs MeOH 3.5% | 0.268 | 0.301 | 0.26265037 |
| MeOH 3.0% vs MeOH 3.5% | 0.301 | 0.301 | 1 |

**
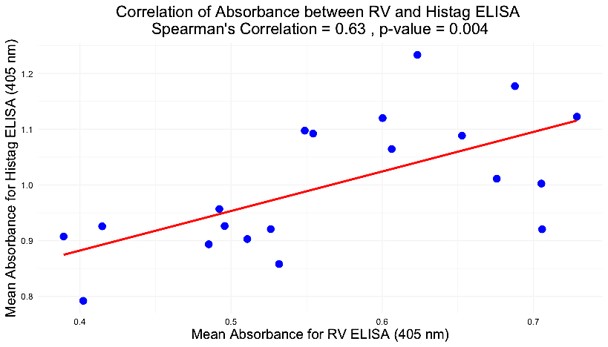
**

**Fig. 1** Spearman correlation analysis assessing the relationship between Histag-ELISA and RV-ELISA rankings derived from the expression analysis of 20 clones secreting DS-1 (G2P[4]) VP8 under the inducible AOX1 promoter.

**
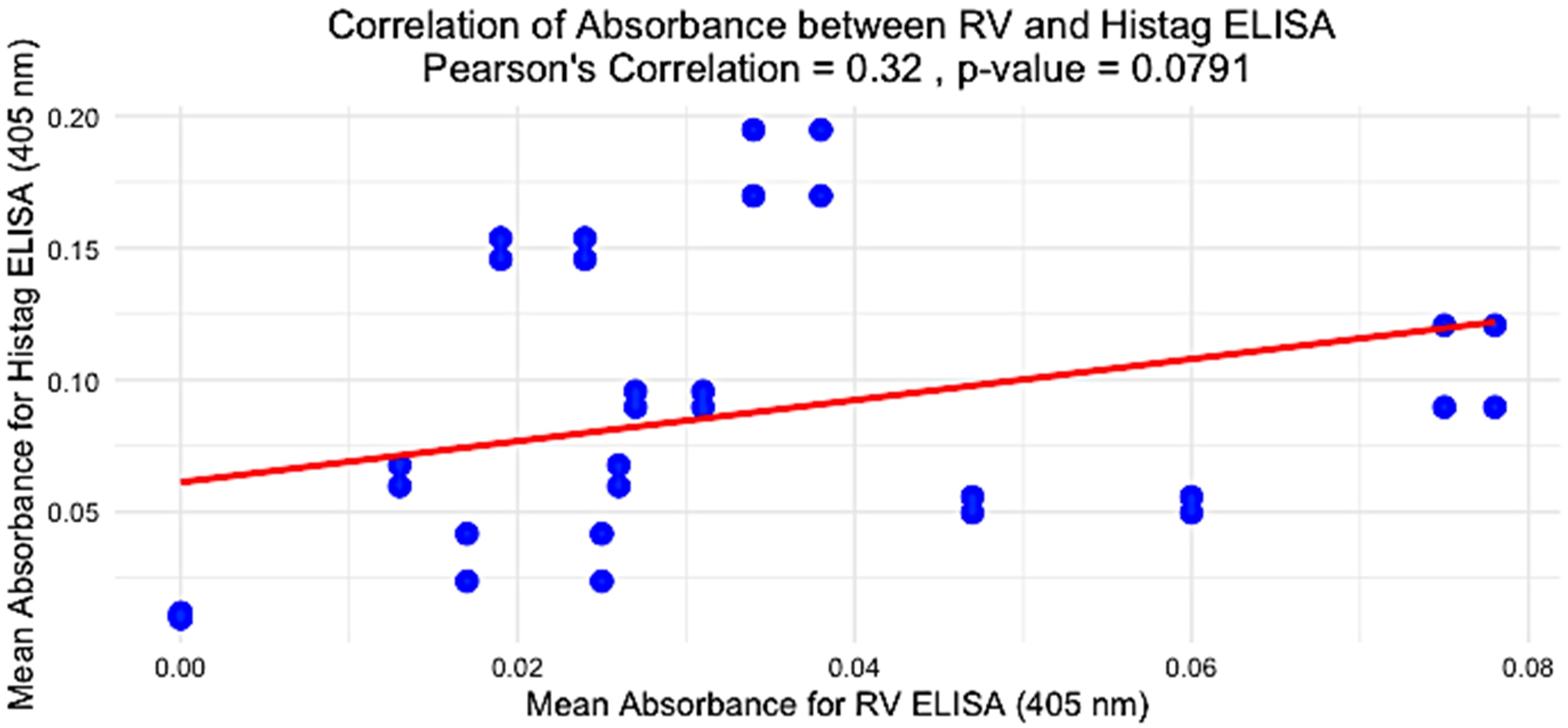
**

**Fig. 2** Pearson correlation analysis assessing the relationship between Histag-ELISA and RV-ELISA across eight distinct methanol induction conditions at OD_600_ 70.

**
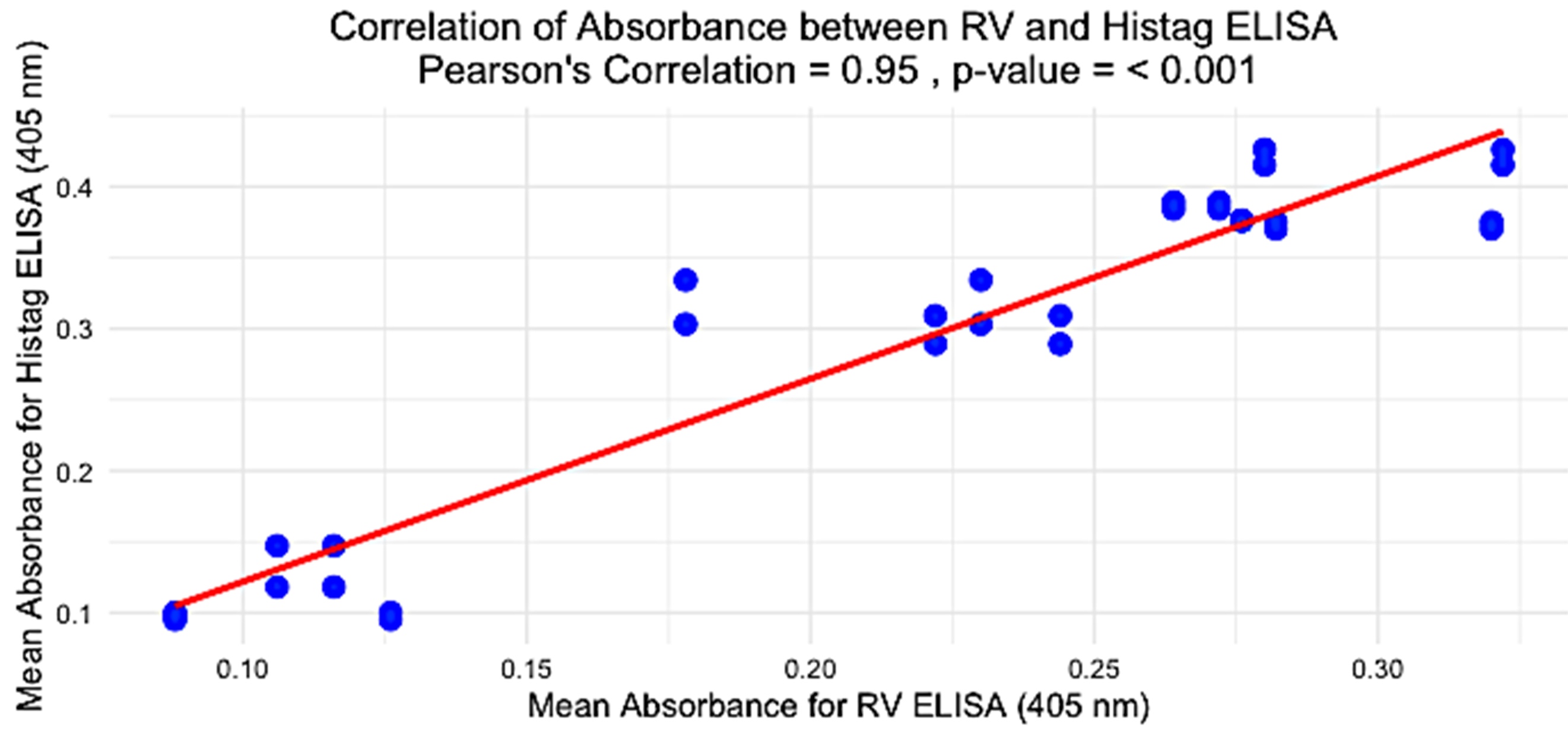
**

**Fig. 2**

**Fig. 3** Pearson correlation analysis assessing the relationship between Histag-ELISA and RV-ELISA across eight distinct methanol induction conditions at OD_600_ 140.

**
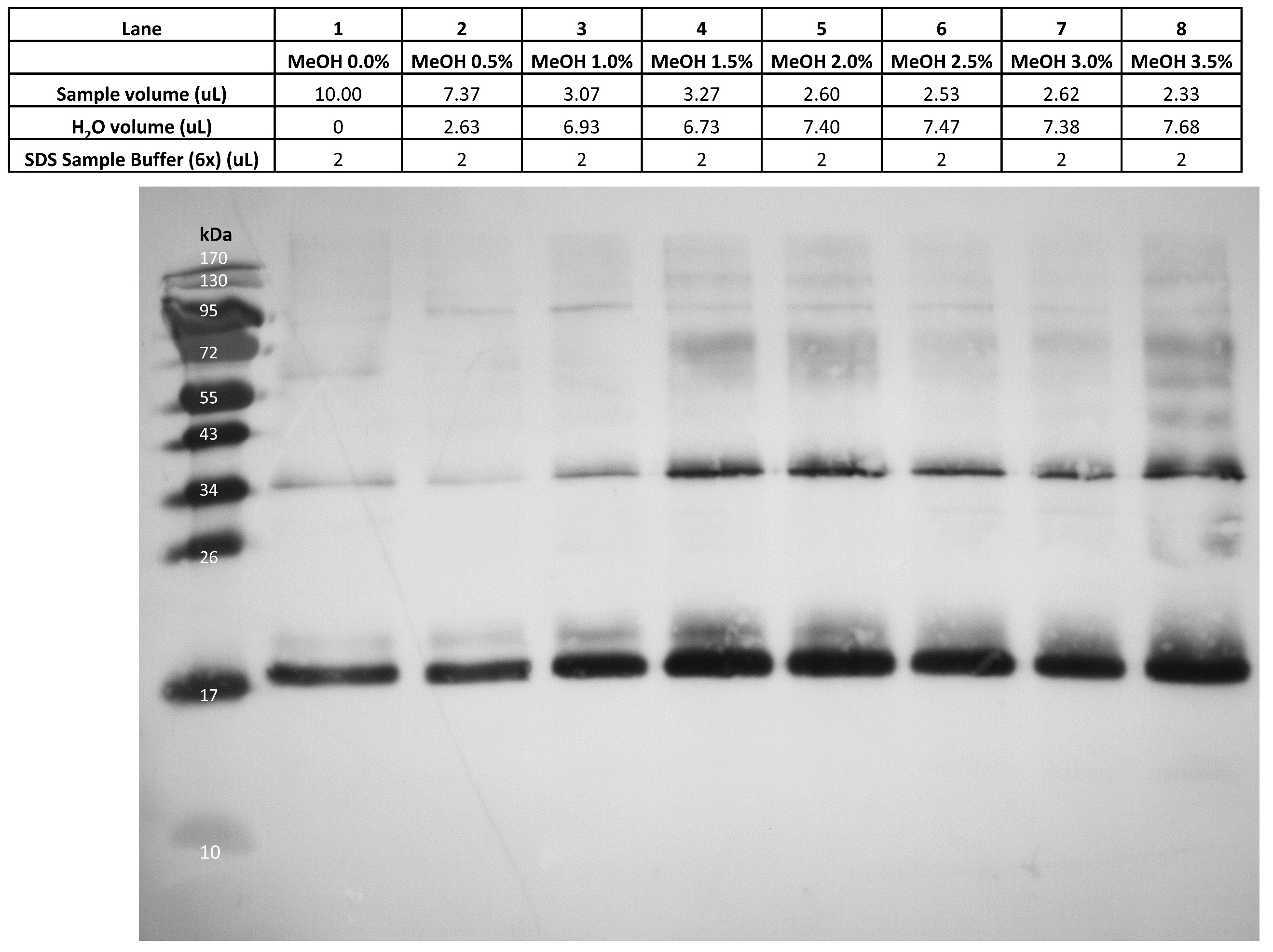
**

**Fig. 4** Western blot using anti-His tag antibody corresponding to the eight methanol induction conditions at OD_600_ 140 (lanes 1–8 correspond to methanol induction concentrations of 0.0%, 0.5%, 1.0%, 1.5%, 2.0%, 2.5%, 3.0%, and 3.5%). Samples were normalized using Histag ELISA signals, and the loaded amounts were determined as indicated in the table above the blot.


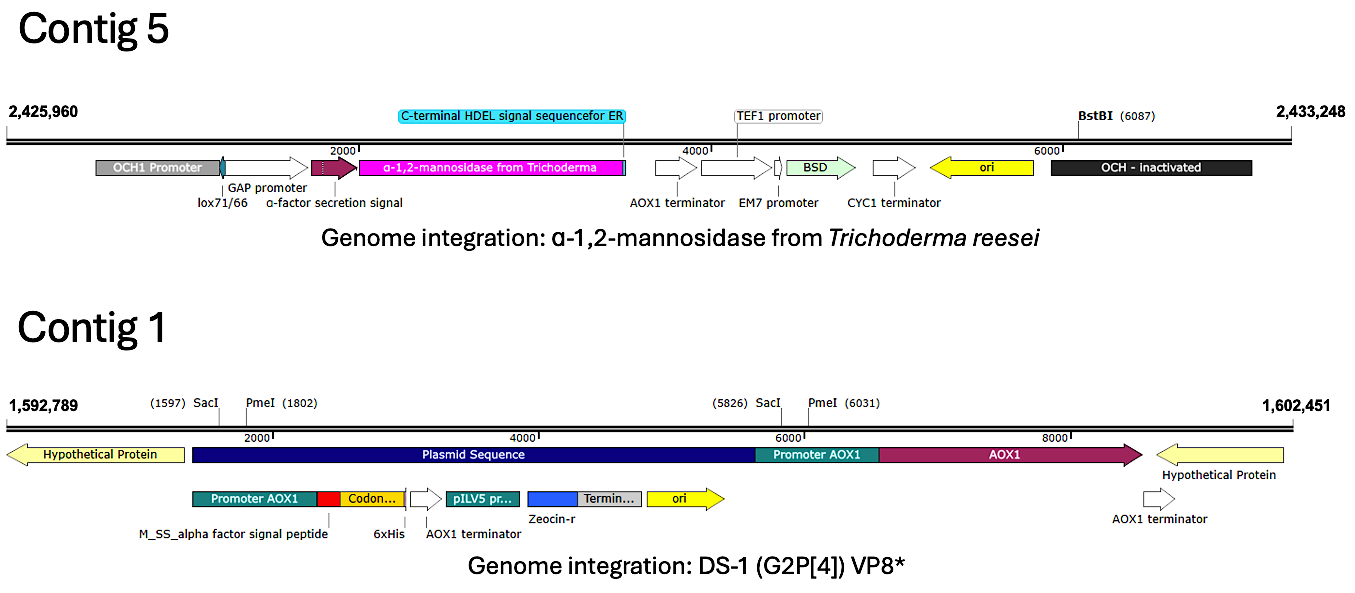


**Fig. 5** Expression cassette schematics illustrating the presence of *T. reesei* α-mannosidase at the *OCH1* locus and the integration of a single copy of the DS-1 (G2P[4]) VP8* gene at the AOX locus confirmed by whole genome sequencing. Whole genome sequencing results and raw data are shown in the file C00015_sample1.zip.

**
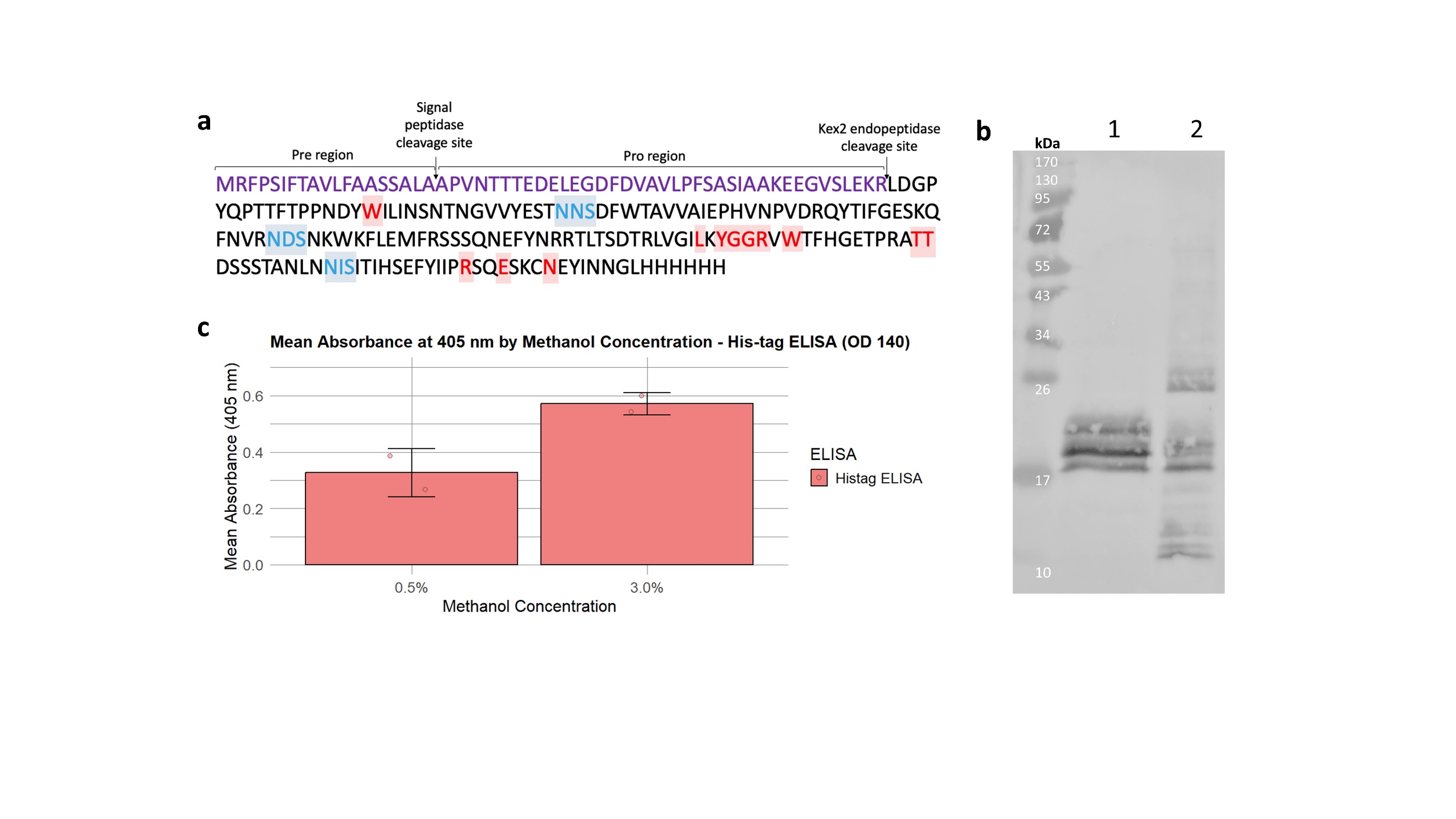
**

**Fig. 6** (a) Protein sequence highlighting key features: the signal peptide with cleavage sites (purple), the truncated WA (G1P[8]) VP8* sequence (black), glycan-binding cavity residues (red) as identified by (Sun et al., 2020), and potential N-linked glycosylation motifs (blue) predicted using the NetNGlyc tool (Steentoft et al., 2013). (b) Indirect ELISA using anti-His to assess WA (G1P[8]) VP8* expression under two distinct methanol induction conditions (0.5% and 3.0%) at OD_600_ 140. (c) Western blot corresponding to the two methanol induction conditions at OD_600_ 140, using an anti-His primary antibody for detection (lanes 1 and 2 correspond to methanol induction concentrations of 0.5% and 3.0%, respectively). Samples were normalized, and 300 ng was loaded into each well.


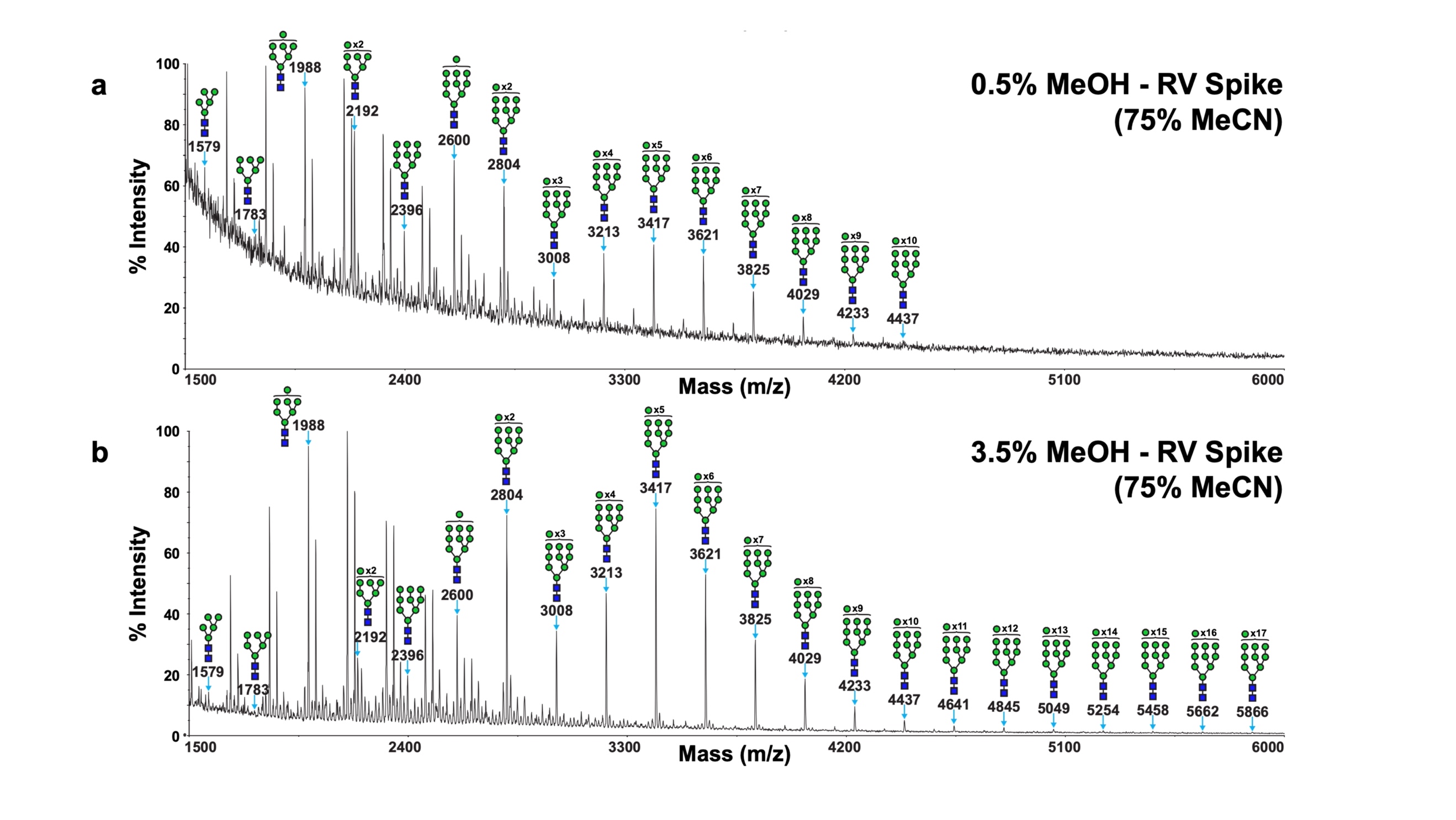


**Fig. 7** (a) MALDI-TOF MS profile of permethylated N-linked glycans derived from SuperMan5 *P. pastoris*-produced DS-1 (G2P[4]) VP8* at 0.5% methanol induction. (d) MALDI-TOF MS profile of permethylated N-linked glycans from the same protein expressed at 3.5% methanol induction. Data were obtained from the 75% MeCN fraction from a C_18_ Sep-Pak. All molecular ions represent the singly charged and sodiated form [M + Na]^+^. Structures shown with brackets have not had their antennal location unequivocally defined.


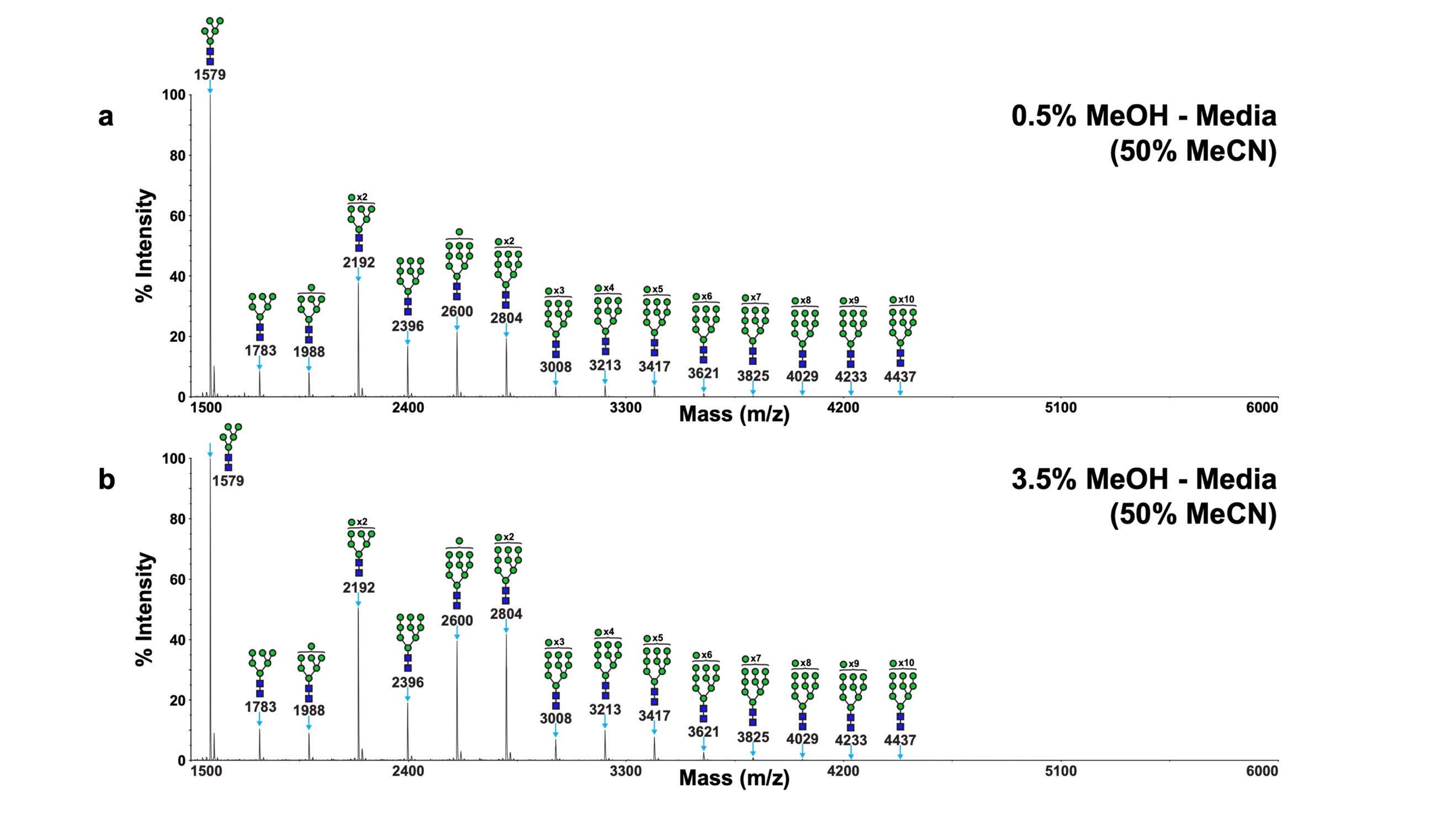


**Fig. 8** (a) MALDI-TOF MS profile of permethylated N-linked glycans derived from the supernatant of SuperMan5 *P. pastoris* expressing DS-1 (G2P[4]) VP8* at 0.5% methanol induction. (b) MALDI-TOF MS profile of permethylated N-linked glycans from the supernatant at 3.5% methanol induction. Data were obtained from the 50% MeCN fraction from a C_18_ Sep-Pak. All molecular ions represent the singly charged and sodiated form [M + Na]^+^. Structures shown with brackets have not had their antennal location unequivocally defined.


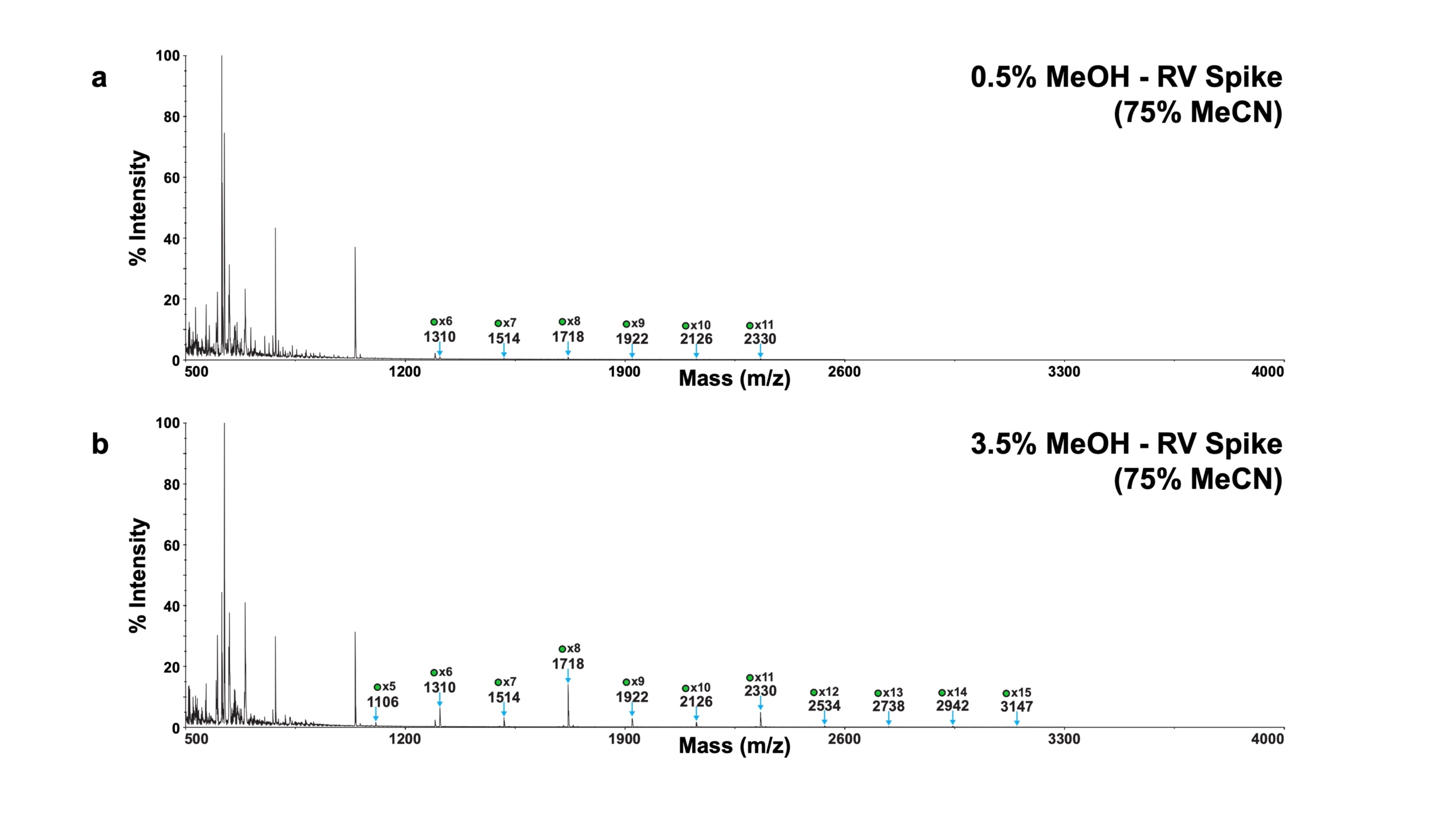

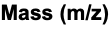


**Fig. 9** (a) MALDI-TOF MS profile of permethylated O-linked glycans from DS-1 (G2P[4]) VP8* at 0.5% methanol induction. (b) MALDI-TOF MS profile of permethylated O-linked glycans from the same protein expressed at 3.5% methanol induction. Data were obtained from the 75% MeCN fraction from a C_18_ Sep-Pak. All molecular ions represent the singly charged and sodiated form [M + Na]^+^.


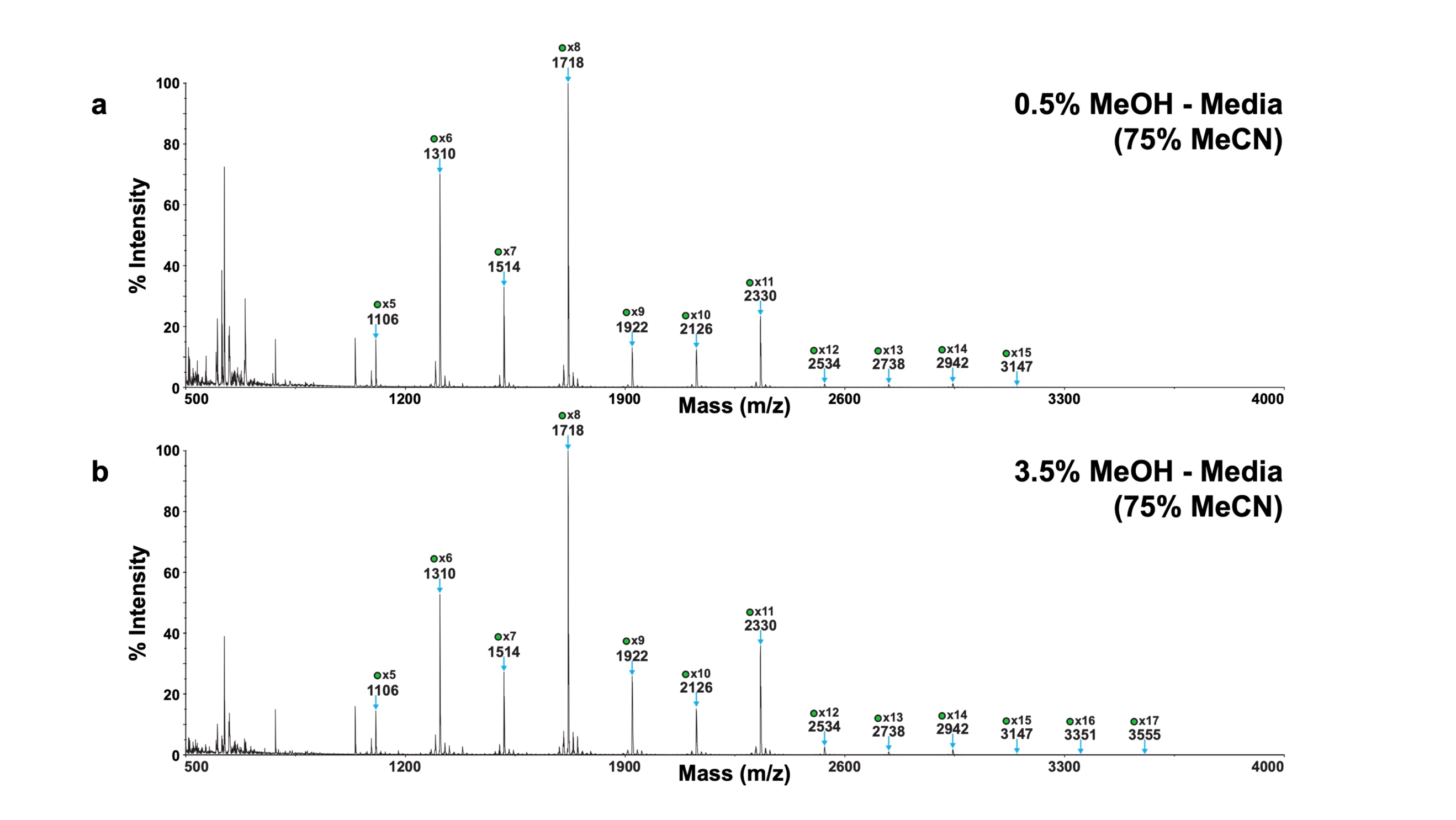


**Fig. 10** (a) MALDI-TOF MS profile of permethylated O-linked glycans from the supernatant of SuperMan5 *P. pastoris* expressing DS-1 (G2P[4]) VP8* at 0.5% methanol induction. (b) MALDI-TOF MS profile of permethylated O-linked glycans from the supernatant at 3.5% methanol induction. Data were obtained from the 75% MeCN fraction from a C_18_ Sep-Pak. All molecular ions represent the singly charged and sodiated form [M + Na]^+^.

**
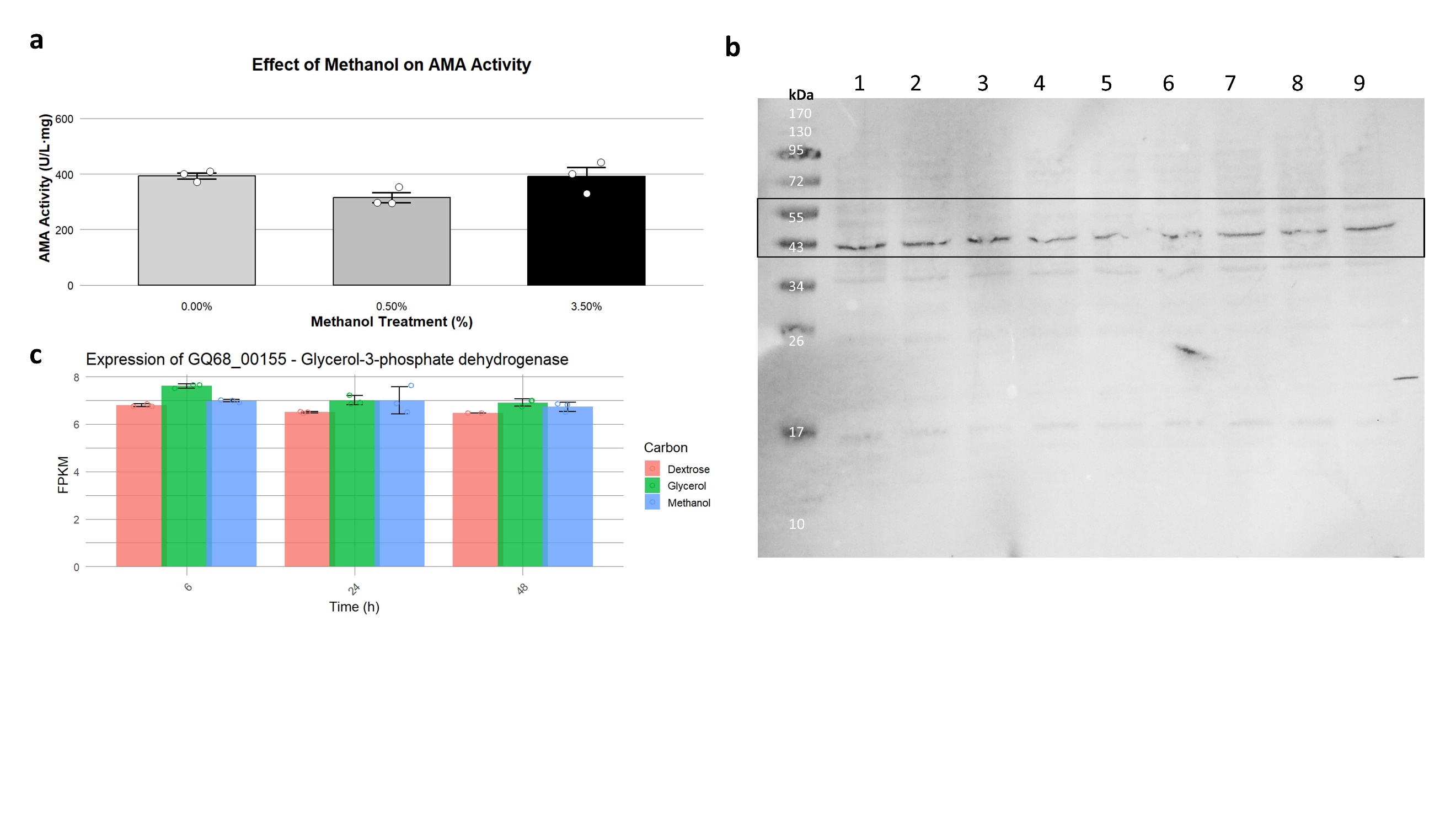
**

**Fig 11.** (a) α-Mannosidase specific activity (AMA) in cell lysates, expressed as Units L⁻¹ mg⁻¹ protein. Values represent means from three biological replicates. (b) Western blot analysis using an anti-HDEL primary antibody. The black box marks the expected molecular weight of *T. reesei* α-1,2-mannosidase. Samples correspond to the same biological replicates used in the AMA assay: lanes 1–3, 0.00% methanol; lanes 4–6, 0.50% methanol; lanes 7–8, 3.50% methanol induction. (c) Expression of the GAP gene, as reported by (Love et al., 2016), under three different carbon sources and time points. Bar chart generated from raw gene expression data provided in Additional file 13: Table S12. The dataset includes log₂ FPKM values and integer read counts for all genes and replicates, originally published by Love et al. (2016) and distributed under the terms of the Creative Commons Attribution 4.0 International License (http://creativecommons.org/licenses/by/4.0/).

**Table 4. Presence and conservation of genes mapped by Love et al. (2016) in the strain engineered in this study**

| **Gene name** | **Description** | **Identity to GS115 genome (parental strain used to engineer Superman5)** |
| --- | --- | --- |
| *ALG1* | Mannosyltransferase, involved in asparagine-linked glycosylation in the endoplasmic reticulum (ER) | 100.00% |
| *ALG11* | Alpha-1,2-mannosyltransferase | 100.00% |
| *ALG12* | Alpha-1,6-mannosyltransferase localized to the ER | 100.00% |
| *ALG3* | Dolichol-P-Man dependent alpha(1-3) mannosyltransferase | 100.00% |
| *ALG9* | Mannosyltransferase, involved in N-linked glycosylation | 100.00% |
| *ANP1* | Subunit of the alpha-1,6 mannosyltransferase complex | 100.00% |
| *BMT1* | Beta-mannosyltransferase involved in cell wall biosynthesis | 100.00% |
| *BMT2* | Beta-mannosyltransferase involved in cell wall biosynthesis | 100.00% |
| *BMT3* | Beta-mannosyltransferase involved in cell wall biosynthesis | 100.00% |
| *BMT4* | Beta-mannosyltransferase involved in cell wall biosynthesis | 100.00% |
| *DPM1* | Dolichol-phosphate mannosyltransferase | 100.00% |
| *GPI14* | Glycosylphosphatidylinositol-alpha 1,4 mannosyltransferase I | 100.00% |
| *HOC1* | Alpha-1,6-mannosyltransferase involved in cell wall mannan biosynthesis | 100% (Truncated as in GS115 ) |
| *KTR4* | Putative mannosyltransferase involved in protein glycosylation | 100.00% |
| *KTR5* | Putative mannosyltransferase involved in protein glycosylation | 100.00% |
| *MNN10* | Subunit of a Golgi mannosyltransferase complex | 100.00% |
| *MNN11* | Subunit of a Golgi mannosyltransferase complex that also contains Anp1p, Mnn9p, Mnn10p, and Hoc1p | 100.00% |
| *MNN2* | Alpha-1,2-mannosyltransferase, responsible for addition of the first alpha-1,2-linked mannose | 100.00% |
| *MNN9* | Subunit of Golgi mannosyltransferase complex also containing Anp1p, Mnn10p, Mnn11p, and Hoc1p | 100.00% |
| *OCH1* | Mannosyltransferase of the cis-Golgi apparatus | Disrupted as described in Supplementary Fig.4 |
| *PAS_chr1-4_0037* | Alpha-1,2-mannosyltransferase | 100.00% |
| *PAS_chr1-1_0286* | Protein O-mannosyltransferase, transfers mannose residues from dolichyl phosphate-D-mannose | 100.00% |
| *PAS_chr1-3_0138* | Alpha-1,2-mannosyltransferase involved in O-and N-linked protein glycosylation | 100.00% |
| *PAS_chr3_0787* | Alpha-1,2-mannosyltransferase | 100.00% |
| *PAS_chr3_0882* | Alpha 1,2-mannosyltransferase involved in glycosyl phosphatidyl inositol (GPI) biosynthesis | 100.00% |
| *PAS_chr3_0215* | Alpha1,2-mannosyltransferase | 100.00% |
| *PAS_chr2-2_0105* | Mannosyltransferase involved in N-linked protein glycosylation | 100.00% |
| *PMT1* | Protein O-mannosyltransferase, transfers mannose residues from dolichyl phosphate-D-mannose | 100.00% |
| *PMT2* | Protein O-mannosyltransferase, transfers mannose residues from dolichyl phosphate-D-mannose | 100.00% |
| *PMT4* | Protein O-mannosyltransferase, transfers mannose residues from dolichyl phosphate-D-mannose | 100.00% |
| *PMT6* | Protein O-mannosyltransferase, transfers mannose from dolichyl phosphate-D-mannose | 100.00% |
| *RPT3* | Alpha 1,2-mannosyltransferase involved in glycosyl phosphatidyl inositol (GPI) biosynthesis | 100.00% |
| *YOR289W* | Protein O-mannosyltransferase, transfers mannose residues from dolichyl phosphate-D-mannose | 100.00% |

**References**

Love, K.R., Shah, K.A., Whittaker, C.A., Wu, J., Bartlett, M.C., Ma, D., Leeson, R.L., Priest, M., Borowsky, J., Young, S.K. 2016. Comparative genomics and transcriptomics of Pichia pastoris. *BMC genomics*, **17**, 1-17.

Steentoft, C., Vakhrushev, S.Y., Joshi, H.J., Kong, Y., Vester‐Christensen, M.B., Schjoldager, K.T.B., Lavrsen, K., Dabelsteen, S., Pedersen, N.B., Marcos‐Silva, L. 2013. Precision mapping of the human O‐GalNAc glycoproteome through SimpleCell technology. *The EMBO journal*, **32**(10), 1478-1488.
